# MicroRNA-16 Restores Sensitivity to Tyrosine Kinase Inhibitors and Outperforms MEK Inhibitors in *KRAS*-Mutated Non-Small Cell Lung Cancer

**DOI:** 10.3390/ijms222413357

**Published:** 2021-12-12

**Authors:** Francesca Fanini, Erika Bandini, Meropi Plousiou, Silvia Carloni, Petra Wise, Paolo Neviani, Mariam Murtadha, Flavia Foca, Francesco Fabbri, Ivan Vannini, Muller Fabbri

**Affiliations:** 1 Immuno-Gene Therapy Factory, IRCCS Istituto Romagnolo per lo Studio dei Tumori (IRST) “Dino Amadori”, 47014 Meldola, Italy; francesca.fanini@irst.emr.it (F.F.); silvia.carloni@irst.emr.it (S.C.); 2 Biosciences Laboratory, IRCCS Istituto Romagnolo per lo Studio dei Tumori (IRST) “Dino Amadori”, 47014 Meldola, Italy; erika.bandini@irst.emr.it (E.B.); meropi.plousiou@irst.emr.it (M.P.); francesco.fabbri@irst.emr.it (F.F.); ivan.vannini@irst.emr.it (I.V.); 3 Department of Microgravity and Translational Regenerative Medicine, Clinic for Plastic, Aesthetic and Hand Surgery, Otto von Guericke University, 39106 Magdeburg, Germany; wisepetra@gmail.com; 4 Extracellular Vesicle Core, The Saban Research Institute, Children’s Hospital Los Angeles, Los Angeles, CA 90027, USA; pneviani@chla.usc.edu; 5 Judy and Bernard Briskin Center for Multiple Myeloma Research, Department of Hematology and Hematopoietic Cell Transplantation, City of Hope, Monrovia, CA 91016, USA; mmurtadha@coh.org; 6 Unit of Biostatistics and Clinical Trials, IRCCS Istituto Romagnolo per lo Studio dei Tumori (IRST) “Dino Amadori”, 47014 Meldola, Italy; flavia.foca@irst.emr.it; 7 Center for Cancer and Immunology Research, Children’s National Hospital, Washington, DC 20010, USA

**Keywords:** microRNAs, tyrosine kinase inhibitors, non-small cell lung cancer, mitogen-activated protein kinases, Kirsten RAS proto-oncogene

## Abstract

Background: Non-small cell lung cancer (NSCLC) is the leading cause of cancer death worldwide. Chemotherapy, the treatment of choice in non-operable cases, achieves a dismal success rate, raising the need for new therapeutic options. In about 25% of NSCLC, the activating mutations of the *KRAS* oncogene define a subclass that cannot benefit from tyrosine kinase inhibitors (TKIs). The tumor suppressor miR-16 is downregulated in many human cancers, including NSCLC. The main objectives of this study were to evaluate miR-16 treatment to restore the TKI sensitivity and compare its efficacy to MEK inhibitors in *KRAS*-mutated NSCLC. Methods: We performed in vitro and in vivo studies to investigate whether miR-16 could be exploited to overcome TKI resistance in KRAS-mutated NSCLC. We had three goals: first, to identify the KRAS downstream effectors targeted by mir-16, second, to study the effects of miR-16 restoration on TKI resistance in KRAS-mutated NSCLC both in vitro and in vivo, and finally, to compare miR-16 and the MEK inhibitor selumetinib in reducing KRAS-mutated NSCLC growth in vitro and in vivo. Results: We demonstrated that miR-16 directly targets the three *KRAS* downstream effectors *MAPK3*, *MAP2K1*, and *CRAF* in NSCLC, restoring the sensitivity to erlotinib in *KRAS*-mutated NSCLC both in vitro and in vivo. We also provided evidence that the miR-16–erlotinib regimen is more effective than the selumetinib–erlotinib combination in *KRAS*-mutated NSCLC. Conclusions: Our findings support the biological preclinical rationale for using miR-16 in combination with erlotinib in the treatment of NSCLC with *KRAS*-activating mutations.

## 1. Introduction

Lung cancer is the main cause of cancer-related death in the world, with an estimated incidence of about 234,000 new cases (both sexes) in the U.S. [1]. Despite all available therapeutic approaches, the 5-year overall survival for lung cancer patients is less than 20% across all stages of the disease [1] and has not significantly improved in the last 40 years. One of the main causes of failure in the treatment of lung cancer is that cancer cells can become resistant to drugs. Therefore, in parallel to searching for new therapeutic options, it is imperative that new strategies to prevent or overcome drug resistance are exploited. Recently, the identification of driving and actionable mutations in lung cancer has led to the development of targeted therapies that block the activation of these mutated genes. Non-small cell lung cancer (NSCLC) represents 80% of lung cancers, and it is estimated that 15% of all NSCLC (especially younger patients with adenocarcinoma histology or a limited smoking history) [2] carry an *EGFR*-activating mutation [3] that increases the responsiveness to EGFR tyrosine kinase inhibitors (TKIs) such as erlotinib, gefitinib, or afatinib [4]. *KRAS* mutations occur in about 25% of NSCLC, are mutually exclusive with *EGFR* mutations [5,6,7], and are related to an intrinsic resistance toward TKIs [8]. Currently, there are no approved targeted therapies for the treatment of *KRAS*-mutated NSCLC, since KRAS targeting has proven pharmacologically challenging and is still considered an “undruggable” protein [9,10]. Recently, one of the strategies to overcome TKI resistance in *KRAS*-mutated NSCLC has been the targeting of the KRAS downstream MAPK effector pathway [6,7,10]. Unfortunately, the use of MEK inhibitors such as trametinib or selumetinib (alone or combined with erlotinib) resulted in limited activity in patients with *KRAS*-mutated NSCLC [11,12]; moreover, the dual inhibition of EGFR and MEK may lead to a secondary activation of the PI3K/AKT pathway [13] or induce MEK activation by CRAF [14].

MicroRNAs (miRNAs) are short (19–24 nucleotide long) noncoding RNA transcripts that inhibit the translation of target mRNAs mostly by binding to their 3′-untranslated region (3′-UTR) [15,16]. MiRNAs control several physiologic functions of cells, and their dysregulation has been described in all types of human cancers (including NSCLC) and several human diseases [17,18,19,20,21,22]. One of the main properties of miRNAs is that each of them has multiple target genes. While one miRNA can reduce the expression of a protein in some cases by only 30–40%, its ability to target several proteins linked in a common pathway may result in profound phenotypical implications [23].

In this study, we identified miR-16 as significantly downregulated in NSCLC compared to the adjacent noncancerous lung tissue counterpart, and we showed that the levels of miR-16 are significantly reduced in *KRAS*-mutated primary NSCLC compared to *KRAS*-nonmutated NSCLC. We provide evidence that miR-16 directly targets the *MAPK* pathway at several levels and that its upregulation restores its sensitivity to erlotinib in *KRAS*-mutated NSCLC both in vitro and in vivo, with results that are better than the TKI–MEK inhibitor combination. These data support a role for miR-16 in overcoming the intrinsic resistance of NSCLC to erlotinib and provide the biological preclinical rationale for a miR-16–erlotinib combination in the treatment of *KRAS*-mutated NSCLC.

## 2. Results

### 2.1. MicroRNA-16 Is Downregulated in NSCLC Primary Samples and Cell Lines

In order to identify miRNAs able to silence genes that are downstream of KRAS and aberrantly activated by the *KRAS* mutations, we performed an in silico analysis of miRNAs predicted to target at least three genes of the *KRAS* pathway and downstream of KRAS by at least three different softwares (TargetScan [24], DIANA miRGen [25,26], and miRanda [27]). We identified miR-16-5p (from now on referred to as miR-16) as the top miRNA predicted to silence *MAPK3*, *MAP2K1*, and *CRAF* (Figure S1). To determine whether miR-16 was downregulated in NSCLC patients, we first collected RNA from 92 paired normal and NSCLC primary samples and assessed the miR-16 expression by qRT-PCR. An inverse relationship was observed between miR-16 levels in cancerous vs. adjacent normal lung tissues (*p* < 0.0001, Figure S2A). Moreover, the inverse relationship of the miR-16 levels between cancerous and adjacent normal lung tissues was maintained within the subgroups of *KRAS* wild-type or *KRAS*-mutated NSCLC primary samples (*p* < 0.0001 and *p* = 0.0025, Figure S2B,C, respectively). Finally, in the cancerous tissue subgroups alone, the miR-16 levels were significantly lower in *KRAS*-mutated vs. *KRAS* wild-type cancers (*p* = 0.0107, Figure S2D). Similarly, the endogenous expression of mir-16 in lung cancer cell lines A549 and NCI-H2009 were significantly lower compared to the total normal lung RNA (#540019, Agilent Technologies) (*p* < 0.05, Figure S3).

### 2.2. MicroRNA-16 Directly Targets MAPK3, MAP2K1, and CRAF

**Figure 1:**
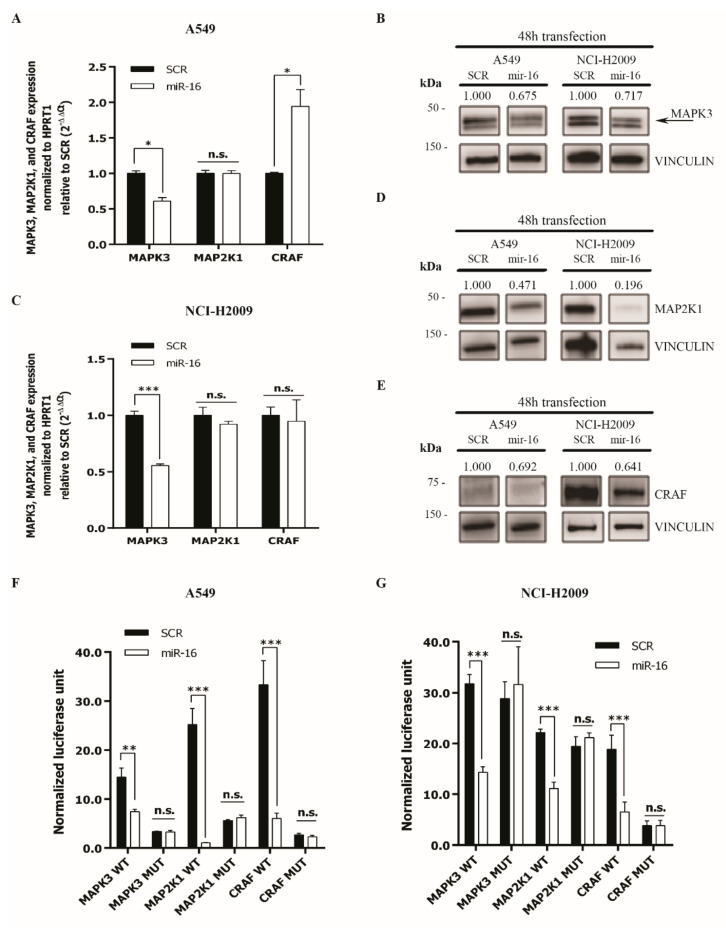
MiR-16 directly targets *MAPK3*, *MAP2K1*, and *CRAF*. (**A**–**E**) mRNA and protein expression levels of MAPK3, MAP2K1, and CRAF in the A549 ((**A**,**B**,**D**,**E**) left) and NCI-H2009 ((**B**–**E**) right) cell lines 48 h after transfection with pre-miR-16 (miR-16) or scrambled (SCR). (**F**,**G**) Luciferase reporter assay in A549 (**F**) or NCI-H2009 (**G**) cells co-transfected for 24 h with constructs containing *MAPK3*, *MAP2K1*, and *CRAF* 3′-UTR wild-type (WT) or mutated (MUT) and miR-16 (or scrambled as a control). The luciferase activity was normalized to the Renilla internal control. Data are expressed as normalized luciferase units and are calculated as the ratio Firefly luciferase activity/Renilla luciferase activity of miR-16-transfected cells normalized to the scrambled of a total of 3 experiments from 3 independent transfections (*n* = 6). Data represent the means ± SD. * *p* < 0.05, ** *p* < 0.01, *** *p* < 0.001, and n.s. = not significant. Multiple *t*-test, corrected for multiple comparisons using the Holm–Sidak method in (**A**,**C**,**F**,**G**).

MiR-16 is predicted to directly target *MAPK3*, *MAP2K1*, and *CRAF* (Figure S1). These kinases are known to be activated by the *KRAS*-amplifying mutation responsible for primary resistance to TKI treatments of NSCLC [28]. To assess whether miR-16 directly targets *MAPK3*, *MAP2K1*, and *CRAF*, we used the A549 and NCI-H2009 cell lines carrying the *KRAS*-activating mutations G12S and G12A, respectively [29,30], and confirmed by home sequencing (Table S1). The A549 and NCI-H2009 cells were transfected with miR-16 (or a scrambled oligonucleotide as the control), and the *MAPK3*, *MAP2K1*, and *CRAF* expression was evaluated. We observed a significant downregulation of *MAPK3* expression in either cell lines, both at the mRNA (*p* < 0.05 in A549 and *p* < 0.001 in NCI-H2009, Figure 1A,C) and the protein levels (Figure 1B and Supplementary Figures S4–S7). In the miR-16-transfected cells, the expression of *MAP2K1* and *CRAF* was not reduced at the mRNA level (Figure 1A,C) but at the protein level after 48 h in both cell lines (Figure 1D,E and Supplementary Figures S8–S15). To assess whether the silencing by miR-16 was direct, we cloned the miR-16-predicted binding sites for *MAPK3*, *MAP2K1*, and *CRAF* downstream of the stop codon of a luciferase gene, and we performed a luciferase reporter assay. We showed that miR-16 reduced the luciferase activity of the reporter plasmid in A549 and NCI-H2009 cells compared to a scrambled miRNA as the control, and this effect was abolished by site-specific deletion/mutagenesis of the miR-16-predicted binding site on the 3′-UTR of the involved genes (Figure 1F,G).

### 2.3. MicroRNA-16 Restores Sensitivity to Erlotinib in KRAS-Mutated NSCLC Cell Lines

**Figure 2:**
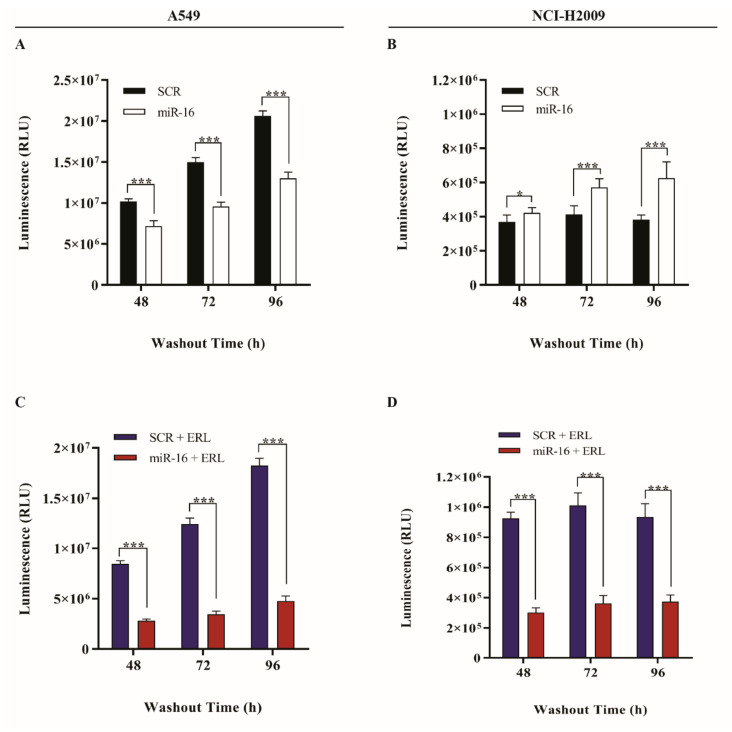
Effects of miR-16 on the sensitivity toward erlotinib in *KRAS*-mutated NSCLC cell lines. (**A**–**D**) CellTiter-Glo^®^ Luminescent Cell viability assay in A549 and NCI-H2009 cells transfected with pre-miR-16 (miR-16) or scrambled (SCR) only (**A**,**B**) or incubated with erlotinib (ERL) for 6 h (**C**,**D**). Experiments were conducted in eight wells and repeated three times. Data represent the means ± SD. * *p* < 0.05 and *** *p* < 0.001. Multiple *t*-test, corrected for multiple comparisons using the Holm–Sidak method in (**A**–**D**).

*KRAS*-activating mutations are the hallmark of primary resistance of NSCLC to TKIs such as erlotinib and gefitinib [28]. In order to assess whether miR-16 is able to overcome the TKIs’ primary resistance by targeting *MAPK3*, *MAP2K1*, and *CRAF* located downstream of the *KRAS*-activated oncogene, we transfected *KRAS*-amplified NSCLC cell lines A549 and NCI-H2009 with the pre-miR-16 mimic or a scrambled miRNA as the control and determined the in vitro cell growth after treatment with erlotinib 1 μg/mL for 6 h, followed by a 48–72–96-h washout. The overexpression of miR-16 was verified by qRT-PCR (Figure S16A,B). The restoration of miR-16 expression significantly reduced the proliferation of the A549 cell line (*p* < 0.001 at 48 h, 72 h, and 96 h, Figure 2A) but not of NCI-H2009 cells (Figure 2B). However, a significant reduction (*p* < 0.001) in the growth of both A549 and NCI-H2009 was observed when the cells were transfected with pre-miR-16 followed by the administration of erlotinib at 48 h, 72 h, and 96 h (Figure 2C,D).

**Figure 3:**
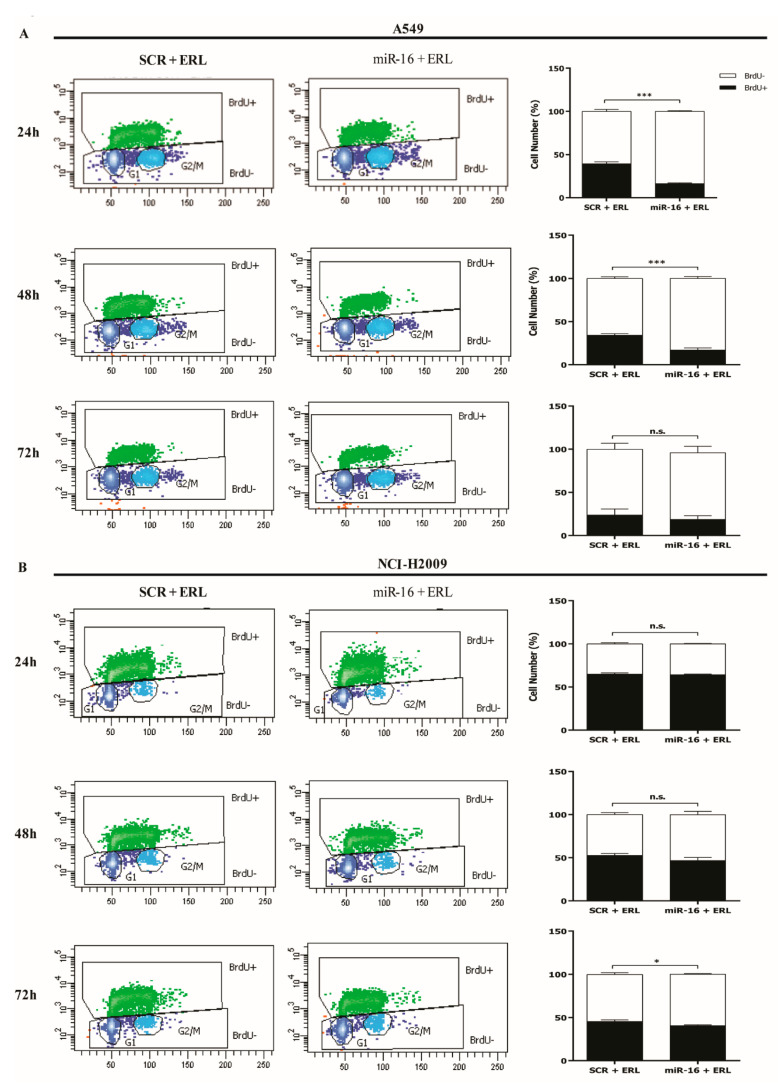
Cytofluorimetric evaluation of erlotinib-induced cell proliferation changes. (**A**,**B**) Representative cytofluorimetric dot plots of proliferating A549 (**A**) and NCI-H2009 (**B**) cells following BrdU incorporation and anti-BrdU antibody incubation from one of the three performed experiments. BrdU (Bromodeoxyuridine) is a thymidine analog that is readily incorporated into newly synthesized DNA by cells progressing through the DNA S phase (DNA synthesis) and cell cycle, which results in BrdU-positive cells (BrdU+). The average percentage resulting from three different experiments is presented in the histograms on the right. All data represent the means ± SD. * *p* < 0.05, *** *p* <0.001, and n.s. = not significant. Multiple *t*-test, corrected for multiple comparisons using the Holm–Sidak method in (**A**,**B**).

In order to quantify the cell growth changes, we first evaluated apoptosis by performing the Annexin V assay. Both cell lines were transfected with pre-miR-16 or scrambled and treated with erlotinib 1 μg/mL for 6 h, followed by 24–48–72-h washout. No changes in the proportion of apoptotic cells were found in either cell line when transfected with pre-miR-16 and erlotinib compared to the scrambled (Figure S17A,B). Since the EGFR pathway is also a main regulator of cell proliferation, we reasoned that the BrdU assay could be more suitable to detecting any possible effects of miR-16 on cancer cell proliferation. We observed a statistically significant reduction of the cell proliferation in A549-transfected cells with pre-miR-16 and treated with erlotinib compared to those scrambled at the 24-h and 48-h washouts (*p* < 0.001, Figure 3A) and in NCI-H2009 cells at the 72-h washout (*p* < 0.05, Figure 3B). No statistically significant differences were observed at the other time points.

### 2.4. MicroRNA-16 Restores Sensitivity to Erlotinib by Targeting MAPK3 and MAP2K1

**Figure 4:**
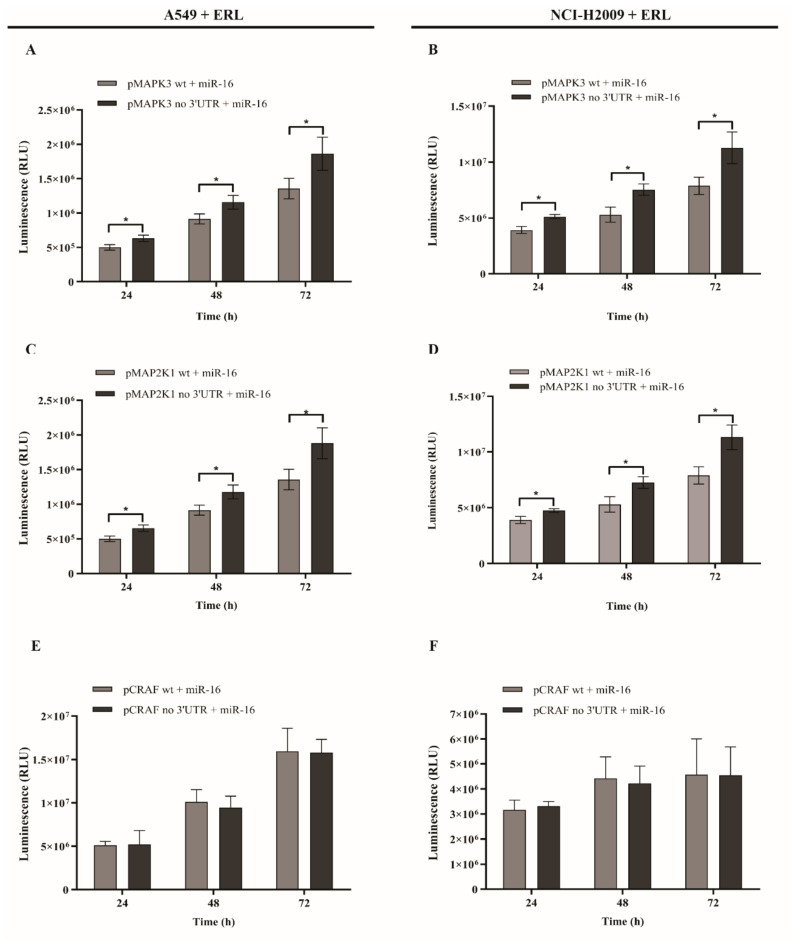
MicroRNA-16 restores the sensitivity to erlotinib by targeting *MAPK3* and *MAP2K1*. (**A**–**F**) The CellTiter-Glo^®^ Luminescent Cell viability assay on A549 cells (**A**,**C**,**E**) and NCI-H2009 cells (**B**,**D**,**F**) after co-transfection with plasmids expressing *MAPK3* (pMAPK3, **A**,**B**)*, MAP2K1* (pMAP2K1; **C**,**D**), or *CRAF* (pCRAF; **E**,**F**) with (wt) or without the 3′-UTR (no 3′-UTR) and miR-16 treated with erlotinib for 6 h. Experiments were conducted in eight wells and repeated three times. Data represent the means ± SD. * *p* < 0.05. Multiple *t*-test, corrected for multiple comparisons using the Holm–Sidak method in (**A**–**F**).

We have shown that miR-16 directly targets *MAPK3*, *MAP2K1*, and *CRAF* (Figure 1F,G). In order to assess whether the effects of miR-16 on TKI sensitivity were mediated by its targeting of these three genes, we performed a rescue experiment. A549 and NCI-H2009 cells were transfected with plasmids expressing *MAPK3*, *MAP2K1*, or *CRAF* without the 3′-UTR, therefore released from the miR-16-targeting effect, and co-transfected with pre-miR-16 and erlotinib. As shown in Figure 4A–D, the growth of both A549 and NCI-H2009 cells was significantly increased (*p* < 0.001) when *MAPK3* and *MAP2K1* with no 3′-UTR were transfected even in presence of miR-16 and erlotinib, reversing the previously shown growth inhibition observed with the miR-16–erlotinib combination. Conversely, no growth difference was detected in cells transfected with the plasmid expressing *CRAF* without 3′-UTR (Figure 4E,F). Overall, these data suggest that the effect of miR-16 on NSCLC cell primary resistance to erlotinib is, at least in part, mediated by its direct targeting of *MAPK3* and *MAP2K1* but not of *CRAF*.

### 2.5. MicroRNA-16 Outperforms Selumetinib in Reducing NSCLC Growth In Vitro and In Vivo

**Figure 5:**
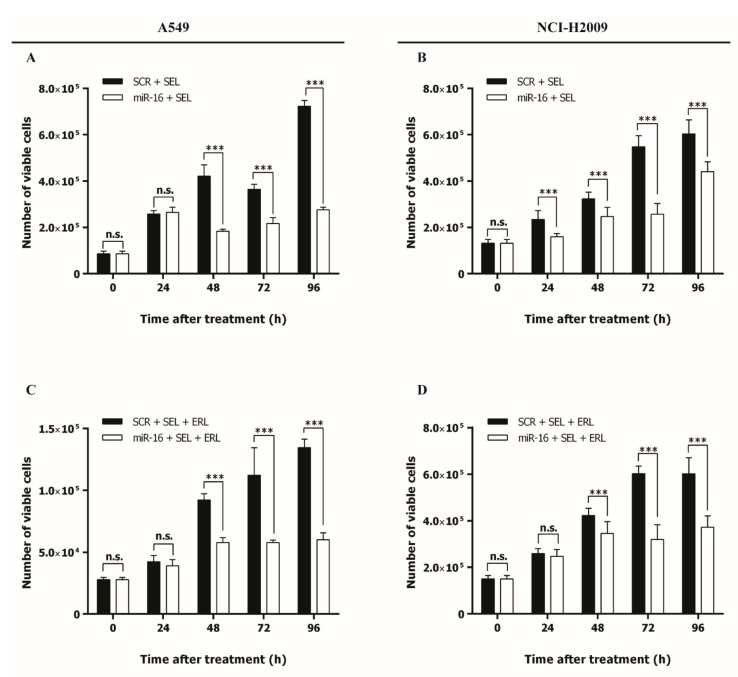
Effect of miR-16 on the activity of selumetinib in *KRAS*-mutated NSCLC cell lines. (**A**–**D**) A Trypan blue exclusion assay to evaluate the cell growth of A549 (**A**,**C**), and NCI-H2009 (**B**,**D**) cells transfected with pre-miR-16 (miR-16) or the scrambled (SCR), then incubated with selumetinib (SEL) continuously alone (**A**,**B**) or in combination with erlotinib (ERL) for 6 h (**C**,**D**). Experiments were conducted in six wells and repeated three times. For every time point, three independent countings were performed. Data represent the means ± SD. *** *p* < 0.001 and n.s. = not significant. Multiple *t*-test, corrected for multiple comparisons using the Holm–Sidak method in (**A**–**D**).

**Figure 6:**
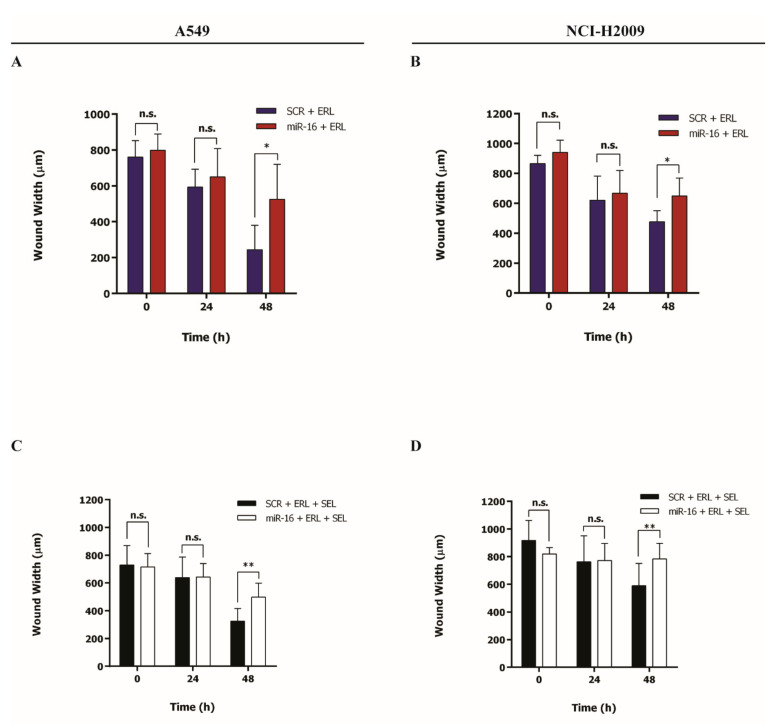
Effects of the miR-16–erlotinib and miR-16–erlotinib–selumetinib combinations on cell migration. (**A**–**D**) Cell culture wound-healing assay to assess the cell migration of A549 (**A**,**C**) and NCI-H2009 (**B**,**D**) cells transfected with pre-miR-16 (miR-16) or the scrambled (SCR), then incubated with erlotinib (ERL) for 6 h alone (**A**,**B**) or in combination with selumetinib (SEL) continuously (**C**,**D**). Experiments were conducted in six wells and repeated three times. For every time point, three independent wound measures were performed. Data represent the means ± SD. * *p* < 0.05, ** *p* < 0.01, and n.s. = not significant. Multiple *t*-test, corrected for multiple comparisons using the Holm–Sidak method in (**A**–**D**).

Selumetinib is a clinically approved potent, selective, and ATP uncompetitive inhibitor of MEK 1/2 kinase [31], previously tested alone or in combination with erlotinib in the treatment of NSCLC [11,12]. Since we have shown that miR-16 silences *MAPK* genes, we directly compared the effects of miR-16 and selumetinib on NSCLC growth. A549 and NCI-H2009 cells were transfected with pre-miR-16 or a scrambled control, and the in vitro cell growth was assessed for up to 96 h after treatment with selumetinib only or in combination with erlotinib. In both cell lines, the miR-16–selumetinib combination significantly reduced NSCLC cell growth compared to the scrambled–selumetinib (*p* < 0.001 after 48 h and 24 h in A549 and NCI-H2009, respectively, Figure 5A,B). Moreover, the addition of miR-16 to the combination of selumetinib and erlotinib significantly inhibited cancer cell growth compared to the scrambled after 48 h (*p* < 0.001, Figure 5C,D). Migration is an essential feature of live cells and is involved in pathological processes such as cancer metastasis and invasion. To further investigate how miR-16 induces its antiproliferative effect in combination with erlotinib or erlotinib plus selumetinib, we studied NSCLC migration. A549 and NCI-H2009 cells were transfected with pre-miR-16 or a scrambled control, and an in vitro cell culture wound closure assay was assessed for up to 48 h after treatment with erlotinib only or in combination with selumetinib. In both cell lines, the miR-16–erlotinib combination significantly inhibited NSCLC cell migration compared to scrambled–erlotinib at 48 h (*p* < 0.05, Figure 6A,B). Moreover, the addition of miR-16 to the combination of erlotinib and selumetinib significantly inhibited cancer cell migration compared to the scrambled after 48 h (*p* < 0.01, Figure 6C,D and Figure S18A,B).

**Figure 7:**
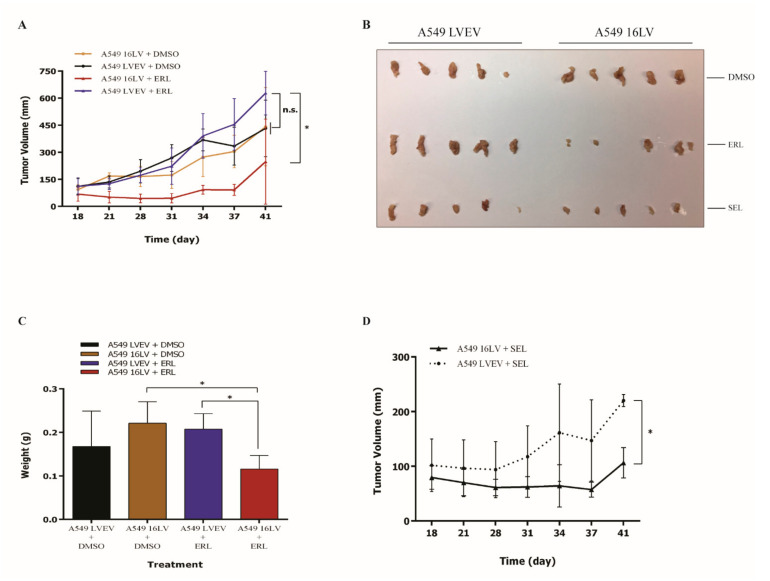
Effect of miR-16 in vivo. Fifteen NSG mice were injected subcutaneously with A549 cells stably expressing miR-16 by lentiviral infection (A549 16LV) or with its empty counterpart (A549 LVEV) and treated with dimethyl sulfoxide (DMSO, *n* = 5/group), erlotinib (ERL, *n* = 5/group), or selumetinib (SEL, *n* = 5/group). Tumor volume was measured for up to 41 days. (**A**) Tumor volume growth of A549 16LV or A549 LVEV xenografts from mice treated with erlotinib (A549 16LV + ERL or A549 16LVEV + ERL) or dimethyl sulfoxide (A549 16LV + DMSO or A549 16LVEV + DMSO). (**B**) Pictures of A549 16LV or A549 LVEV ex vivo xenografts from mice treated with dimethyl sulfoxide (DMSO), erlotinib (ERL), or selumetinib (SEL) at day 41, when animals were euthanized. (**C**) Tumor weights of A549 16LV or A549 LVEV xenografts from mice treated with erlotinib (A549 16LV + ERL or A549 16LVEV + ERL) or dimethyl sulfoxide (A549 16LV + DMSO or A549 16LVEV + DMSO) at day 41, when animals were euthanized. (**D**) Tumor volume of A549 16LV or A549 LVEV xenografts from mice treated with selumetinib (A549 16LV + SEL or A549 16LVEV + SEL). Data represent the means ± SD. * *p* < 0.05 and n.s. = not significant. Multiple *t*-test, corrected for multiple comparisons using the Holm–Sidak method in (**A**,**C**,**D**).

These in vitro data prompted us to test the in vivo effects of miR-16 on NSCLC growth alone or during treatment with erlotinib or selumetinib. We generated A549 stably expressing miR-16 by lentiviral infection (A549-16LV) or its empty counterpart (A549-LVEV). Fifteen NSG mice were injected subcutaneously with A549-16LV (Group A) and 15 mice with A549-LVEV (Group B). Mice of groups A and B were treated with DMSO (*n* = 5/group), erlotinib (*n* = 5/group), or selumetinib (*n* = 5/group), and the tumor volumes were measured for up to 41 days. A statistically significant reduction in tumor volumes was observed for the group of animals treated with A549-16LV and erlotinib compared to A549-LVEV and erlotinib (*p* < 0.05). No statistically significant differences were observed between the A549-LVEV plus erlotinib group compared to the two groups of animals (A549-LV16 and A549-LVEV) treated with DMSO (Figure 7A,B). Additionally, upon necropsy, the tumor weights were significantly lower in the A549-16LV plus erlotinib group (*p* < 0.05, Figure 7C). Finally, the tumor volumes in mice treated with A549-16LV plus selumetinib were significantly reduced compared to A549-LVEV plus selumetinib at day 41 (*p* < 0.05, Figure 7B,D). Overall, these experiments indicated that the upregulation of miR-16 in combination with erlotinib or selumetinib reduced the NSCLC growth in a xenograft murine model.

## 3. Discussion

TKIs such as erlotinib are the standard therapy for patients with *EGFR*-mutant NSCLC [32], but several studies have supported their use also in *EGFR* wild-type NSCLC [33,34]. *EGFR* mutations are mutually exclusive with *KRAS* mutations. *KRAS*-activating mutations occur in about 25% of NSCLC [5,6,7] and are associated with the development of a primary resistance to TKIs [8], therefore limiting the long-term clinical benefits of this kind of therapy in *KRAS*-mutated NSCLC patients. At present, there are no standard targeted therapies for the treatment of *KRAS*-mutated NSCLC, given that KRAS is still considered an “undruggable” protein whose inhibition is yet pharmacologically challenging [9,10]. Targeting of the *KRAS* downstream *MAPK* effector pathway represents one of the most recent strategies to overcome TKI resistance in *KRAS*-mutated NSCLC [6,7,10]. However, the use of MEK inhibitors such as trametinib or selumetinib (alone or combined with erlotinib) resulted in limited activity in patients with *KRAS*-mutated NSCLC [11,12]. In fact, the concomitant inhibition of EGFR and MEK may lead to a secondary activation of the *PI3K*/*AKT* pathway [13] or induce MEK activation by CRAF [14]. The identification and clinical development of alternative molecules able to restore the sensitivity to TKIs in *KRAS*-mutated NSCLC patients will lead to a new treatment for patients with primary resistance. The silencing of kinases downstream of *KRAS*, such as *MAPK3*, *MAP2K1*, and *CRAF*, could be exploited as a new strategy to reach this goal. MicroRNAs have several features that make them suitable for this kind of purpose. One of the main properties of miRNAs is that each miRNA has multiple target genes and has the ability to target several proteins linked in a common pathway, resulting in a modulation of the entire signaling [23]. There are many studies that have already demonstrated how miRNAs are involved in pathway regulations and how aberrations in the pathway activation are associated with a dysregulated expression of specific miRNAs. For instance, in NSCLC, it has been shown that the let-7 family is responsible for *RAS* silencing at the post-transcriptional level [35], miR-128b targets *EGFR* [36], and miR-7 plays a role as a regulator of both *EGFR* and *CRAF* [37]. More recently, mir-135a-1 was identified as an inhibitor of multiple oncogenic pathways (including *EGFR*) acting as a tumor suppressor in metastatic prostate cancer both in vitro and in vivo [38]. Wang et al. identified a list of miRNA:mRNA interactions that are relevant to the resistance and response to TKI treatment in NSCLC. Specifically, they demonstrated the following miR-target interactions: miR34a:*PLCG1*, miR-30a-5p:*PIK3R2*, miR-27a:*GRB2*, and miR-302b/miR-520e:*JAK1*. Their study concluded that these miRNAs could be effective molecular targets in resistant NSCLC [39]. Several groups are studying the modulation of the *PI3K*/*AKT* pathway as a promising strategy to overcome resistance to *EGFR* pathway-based therapies in NSCLC, considering the several shared downstream effectors common to the two signaling networks. The use of a combination of EGFR and IGF-1R inhibitors, as well as the knockdown of PIK3R2, significantly decreased the phosphorylated AKT (p-AKT) expression. The expression of PIK3R2 is reduced by miR-30a-5p upregulation, leading to NSCLC apoptosis and the inhibition of invasiveness and migration, therefore highlighting a potential role of this miRNA as a novel cancer treatment [40]. Interestingly, miR-7 could reverse vemurafenib resistance in melanoma cell lines that overexpress EGFR, IGF-1R, and CRAF. The administration of miR-7 significantly impairs the *MAPK* and *PI3K*/*AKT* pathways by targeting those genes [41], supporting the strategy of inhibiting the downstream effectors shared by different pathways. Mir-16 has been indicated to be downregulated and to act as a tumor suppressor in several cancers, including NSCLC [42]. The role of mir-16 in modulating the drug resistance was widely investigated in relation to *BCL2* targeting [43,44,45,46], but there is no evidence about a possible role of this miRNA in affecting the primary resistance to TKIs in NSCLC.

**Figure 8:**
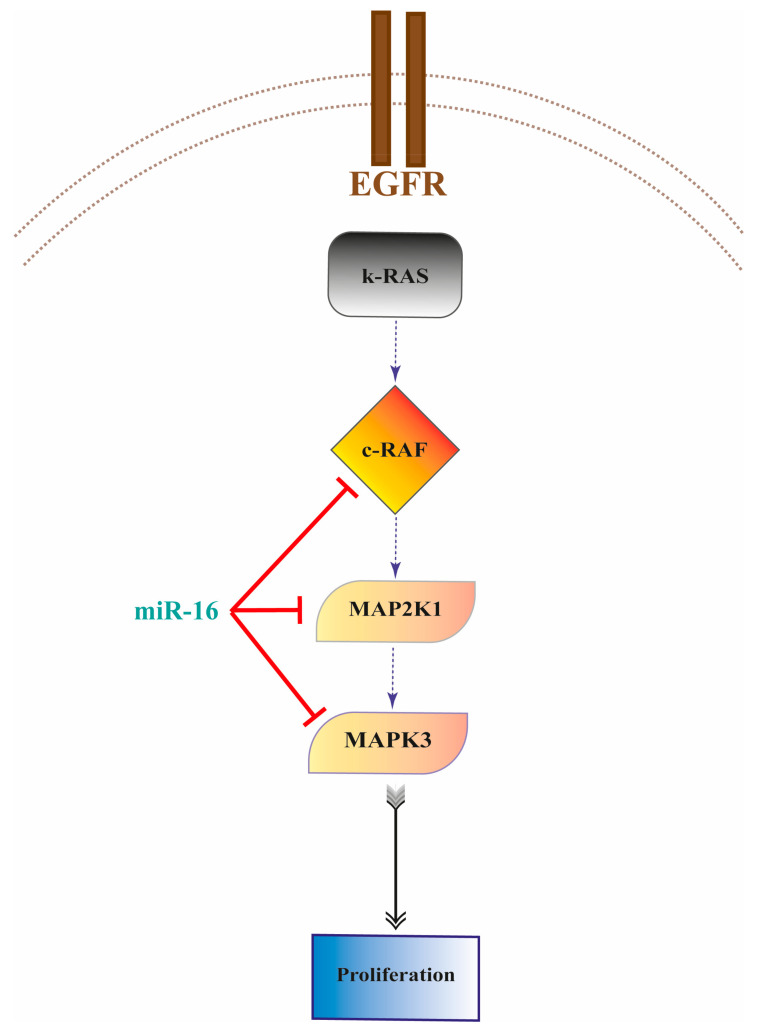
Graphic abstract. MiR-16-5p directly targets c-RAF, MAP2K1 (MEK1), and MAPK3 (ERK1) downstream of K-RAS, therefore reducing the proliferation of NSCLC cell lines carrying K-RAS-activating mutations.

In this study, we also reported miR-16 as significantly downregulated in NSCLC tumor tissues compared to the adjacent lung normal tissue counterparts, and we found that the levels of miR-16 are significantly reduced in *KRAS*-mutated primary NSCLC compared to *KRAS*-nonmutated NSCLC. In addition to the recent evidence that miR-16 exerts its tumor suppressor role by targeting *KRAS* in colorectal cancer [47], we demonstrated that miR-16 directly targets the *MAPK* pathway at several levels and that the silencing operated by it on *MAPK3* and *MAP2K1* (Figure 8) restores its sensitivity to erlotinib in *KRAS*-mutated NSCLC both in vitro and in vivo. Our experiments prompt some elements of discussion. First, we observed different behaviors of A549 and NCI-H2009 cells when miR-16 was overexpressed with no TKI treatment. Such differences disappeared when the cells were treated with the combination of miR-16 and erlotinib. These results could be related to the different type of *KRAS* mutation occurring in the two cell lines; however, further studies to clarify this aspect are warranted. Another consideration was related to the different timings of cell proliferation inhibition between the two cell lines, with A549 being inhibited at 24 h and 48 h and NCI-H2009 at 72 h. Our study also showed that the miR-16 restoration of sensitivity to erlotinib is mediated by its targeting of *MAPK3* and *MAP2K1* but not *CRAF*, despite the fact that all three genes are direct targets of miR-16. It is unclear how the miR-16 silencing of *CRAF* affects NSCLC biology, and future studies should clarify this point.

We also showed that miR-16 increases the NSCLC response to selumetinib both in vitro and in an in vivo murine xenograft model, providing the rationale for the combined administration of the two molecules.

## 4. Materials and Methods

### 4.1. Patient Samples

The total number of patients included in the analyses was 92. All the NSCLC samples were collected at the Morgagni-Pierantoni Hospital of Forlì and stored at IRST IRCCS, Italy. The histological, clinical, and receptor status characteristics are listed in Table S2. All patients provided written informed consent prior to inclusion in the study, and collection of the samples was approved by the Romagna Ethics Committee (C.E.R.O.M. protocol #318/2015 I.5/109, approved on 14 May 2015). In addition, the study was approved by the IRST IRCCS Medical Scientific Committee (CMS protocol #3360/v.3, approved on 2 July 2013).

### 4.2. Cell lines and Cell Cultures

Human lung cancer cell lines A549 and NCI-H2009 were purchased from American Type Culture Collection (Manassas, VA, USA). Human A549 cells were maintained in F-12K (ATCC), supplemented with 10% final concentration of FBS (Gibco, Thermo Fisher Scientific, Waltham, MA, USA), according to the product information sheet. Human NCI-H2009 was maintained in HITES (ATCC-formulated DMEM:F12) and supplemented with a 5% final concentration of FBS (Gibco, Thermo Fisher Scientific) and other components according to the product information sheet. All cell lines were cultured at 37 °C in 5% CO_2_. Each cell line was screened for mycoplasma contamination every month. The *KRAS* mutation status of the cell lines used in this study is summarized in Table S1.

### 4.3. Drugs

Erlotinib was obtained from the IRST IRCCS oncology pharmacy as 100 mg film-coated tablets (Tarceva^®^, Roche Registration Ltd., Basel, Switzerland) and was dissolved in 10 mL sterile dimethyl sulfoxide, DMSO (Cryoserv® Mylan Pharmaceutical, Canonsburg, PA, USA), in order to obtain a stock solution of 10 mg/mL corresponding to 23.26 mM. Exposure was performed at 1 μg/mL for 6 h. Selumetinib was purchased as a powder from MCE (MedChem Express, www.medchemexpress.com (accessed on 8 November 2021)) and resuspended in sterile DMSO (Cryoserv® Mylan Pharmaceutical) in order to obtain a stock concentration of 2.185-mM solution. Exposure was performed at 1 μM continuously.

### 4.4. Transfection with microRNA Mimics

Pre-miRNA miRNA precursor molecules for hsa-miR-16-5p and pre-miR miRNA precursor scrambled negative control #1 (SCR) were purchased from Ambion (Thermo Fisher Scientific). Transfections were performed using 100 nM of the miRNA specific-strand precursor molecules or control and the TransIT-X2^®^ Dynamic Delivery System (Mirus Bio LLC, Madison, WI, USA), according to the manufacturer’s instructions. RNA and proteins were collected at 24–48–72–96 h after transfection, according to the experimental schedule. MiRNA transfection efficiencies were evaluated by real-time qRT-PCR.

### 4.5. In Vitro Proliferation Assay

Cells were seeded in 25 cm^2^ flasks (Greiner Bio-One, Kremsmünster, Austria) and allowed to attach for 24 h before transfection. Four hours after transfection with pre-miR hsa-miR-16-5p, A549 and NCI-H2009 cells were treated with erlotinib at a single concentration of 1 μg/mL for 6 h. Cells were then harvested, plated in 96-well flat-bottomed microtiter plates, and subjected to 24–48–72–96 h washout, depending on schedule treatment. At each exposure time, the viability test was performed by the CellTiter-Glo**^®^** Luminescent Cell Viability Assay (Promega, Madison, WI, USA), as described below. All experiments were performed in eight wells and repeated at least three times.

### 4.6. In Vitro Cell Growth Assays

A549 and NCI-H2009 cells were seeded into six-well plates and allowed to attach for 24 h before transfection. After transfection, dependent on the experimental group, cells were treated as follows:

- 4 h post-transfection with erlotinib at a single concentration of 1 μg/mL for 6 h and, following a medium change, allowed for 24–48–72–96 h washout;
- 10 h post-transfection with selumetinib at a single concentration of 1 μM continuously for 24–48–72–96 h.

At the end of every time point, cells were harvested and counted using the Trypan blue exclusion assay. Experiments were carried out three times independently, and for every time point, three independent counts were performed.

### 4.7. In Vitro Cell Migration Assays

A cell culture wound closure assay [48] was performed to evaluate the A549 and NCI-H2009 cells’ migration ability after transfection with pre-miR hsa-miR-16-5p followed by treatment with erlotinib at a single concentration of 1 μg/mL alone or in combination with selumetinib at a single concentration of 1 μM. Briefly, 150 × 10^5^ cells were seeded into 6-well plates and allowed to attach for 24 h before transfection. After transfection, dependent on the experimental group, cells were treated as follows:

- 4 h post-transfection with erlotinib at a single concentration of 1 μg/mL for 6 h and, following a medium change, allowed for 24–48 h washout;
- 10 h post-transfection after erlotinib treatment with selumetinib at a single concentration of 1 μM continuously for 24–48 h.

The day after a vertical winddown, a 70–80% confluence monolayer was made with a 200 μL pipette tip (Finntip Pipette Tips, Thermo Fisher Scientific). By the use of an inverted microscope Olympus IX51 (Olympus Italia S.r.l., Segrate, MI, Italy) connected to a Nikon Digital Sight DS-Vi1 camera, a snapshot of the wound was taken at a 4× magnification (UPlanFI 4×/0.13 lens) in order to check its closure at the initial time (0 h) and then every 24 h. The analysis of the results was made by Nikon NIS-Elements D Software measuring the distance of one side of the scratch to the other using a scale bar. Experiments were carried out three times independently, and for every time point, three independent measures were estimated.

### 4.8. DNA, RNA, and Protein Extraction

Genomic DNA was isolated using the QIAamp DNA Mini Kit (Qiagen, Milan, Italy), according to the manufacturer’s instructions. The DNA quantity was assessed with NanoDrop ND-1000 (Thermo Fisher Scientific, Waltham, MA, USA). RNA was isolated using the mirVana™ miRNA Isolation Kit (Ambion, Thermo Fisher Scientific, Waltham, MA, USA), according to the manufacturer’s instructions. The RNA quantity was assessed with NanoDrop ND-1000 (Thermo Fisher Scientific, Waltham, MA, USA). The total protein extracts were prepared in ice-cold RIPA lysis buffer 1∗ (Santa Cruz Biotechnology, Dallas, TX, USA) containing 10 μl PMSF solution, 10 μL sodium orthovanadate solution, and 15 μL protease inhibitor cocktail per ml of 1∗ RIPA lysis buffer. The protein quantity was assessed with the PierceTM BCA Protein Assay Kit (Thermo Fisher Scientific, Waltham, MA, USA).

### 4.9. Analysis of KRAS Mutation Status

The primers used for genomic DNA amplification and sequencing are reported in Table S3 and were previously published by Kypuy et al. [49]. The reaction mixture was made up using Takara Ex Taq (Takara Biotechnology, Kusatsu, Shiga, Giappone) and consisted of 100 ng of genomic DNA, 5 μL of 10∗ Ex Taq Buffer, 4 μL of dNTP Mix (2 mM), 0.5 μL of Takara Ex Taq DNA polymerase (1 unit), 2 μL of each primer, and 26.5 μL of not DEPC-treated nuclease-free water (Ambion, Thermo Fisher Scientific, Waltham, MA, USA). PCR cycling was performed on the DNA Engine PTC-200 Peltier Thermal Cycler (MJ Research). The 189-bp amplicon was run according to the following conditions: one cycle of 95 °C for 5 min; 40 cycles of 95 °C for 15 s, 56 °C for 15 s, and 72 °C for 15 s; and one cycle of 72 °C for 3 s. The 92-bp amplicon was run according to the following conditions: one cycle of 95 °C for 5 min; 40 cycles of 95 °C for 15 s, 58 °C for 15 s, and 72 °C for 15 s; and one cycle of 72 °C for 3 s. The size and integrity of all PCR products were confirmed on 2% agarose gels. PCR products were then purified using the Minielute PCR purification kit (Qiagen, Milan, Italy) and then submitted to sequencing using the BigDye Terminator 3.1 Reaction Cycle Sequencing kit (Applied Biosystems, Thermo Fisher Scientific, Waltham, MA, USA). Sequence reactions were purified using the DyeEx 2.0 Spin kit (Qiagen, Milan, Italy) and separated by capillary electrophoresis with laser-induced fluorescence detection (ABI-3130 Genetic Analyzer, Applied Biosystems, Thermo Fisher Scientific, Waltham, MA, USA).

### 4.10. Evaluation of miRNAs and mRNA Expression

MiR-16-5p expression was evaluated using TaqMan miRNA Assays (Applied Biosystems, Thermo Fisher Scientific, Waltham, MA, USA). Briefly, complementary DNA (cDNA) was synthesized using 10 ng of RNA as a template, gene-specific stem–loop Reverse Transcription primers, and the TaqMan microRNA Reverse Transcription kit (Applied Biosystems, Thermo Fisher Scientific, Waltham, MA, USA). MAPK3, MAP2K1, and CRAF expression was evaluated using TaqMan Gene Expression Assays (Applied Biosystems, Thermo Fisher Scientific, Waltham, MA, USA). Briefly, complementary DNA (cDNA) was synthesized using 80 ng of RNA as a template and the TaqMan RT PCR kit (Applied Biosystems, Thermo Fisher Scientific, Waltham, MA, USA). Real-time qRT-PCR was carried out in an Applied Biosystems 7500 Real-Time PCR System (Thermo Fisher Scientific, Waltham, MA, USA) using cDNA, TaqMan probe, and TaqMan universal PCR master mix (Applied Biosystems, Thermo Fisher Scientific, Waltham, MA, USA). Experiments were performed in triplicate and normalized to small nuclear RNU6B or RNU48 or HPRT1, which were used as the internal controls. The relative expression levels were calculated using the comparative Ct method (ΔΔC_t_).

### 4.11. Protein Expression Analysis

Western blotting was used to confirm the expression of MAPK3, MAP2K1, and CRAF in pre-miR-16-5p-transfected cells. Total proteins, in an amount ranging from 20 to 100 μg, were electrophoresed in Criterion TGX Stain-Free Precast Gel 4–20% (Bio-Rad Laboratories, Hercules, CA, USA) and electroblotted onto pure PVDF membranes (Trans-Blot Transfer Turbo midi-format 0.2 μm; Bio-Rad Laboratories, Hercules, CA, USA) using the Trans Blot Turbo System (Bio-Rad Laboratories, Hercules, CA, USA). Primary antibodies and dilutions were as follows: P44/p42 MAPK (Erk1/2) Antibody (#9102 Cell Signaling Technology, Danvers, MA, USA) 1:1000, MEK1 (61B12) Mouse mAb (#2352 Cell Signaling Technology, Danvers, MA, USA) 1:2000, CRAF Antibody (#9422 Cell Signaling Technology, Danvers, MA, USA) 1:1000, and Vinculin clone FB11 (IgG1) monoclonal Ab (Biohit Healthcare, Helsinki, Finland) 1:1000. Secondary antibodies and dilutions were as follows: Rabbit IgG-heavy and light-chain Antibody HRP-conjugated (Bethyl Laboratories, Montgomery, TX, USA) 1:10,000, Mouse IgG-heavy and light-chain Antibody HRP-conjugated (Bethyl Laboratories, Montgomery, TX, USA) 1:10,000, and Precision Plus Protein Western C StrepTactin–HRP conjugate (Bio-Rad Laboratories, Hercules, CA, USA) 1:10,000. Membrane blocking and antibody incubation were performed according to the Cell Signaling Western Immunoblotting protocol. The Clarity Western ECL substrate (Bio-Rad Laboratories, Hercules, CA, USA) was used to detect antigen–antibody reactions. The bands were quantified with ImageJ 1.50i Software (https://imagej.nih.gov/ij/index.html (accessed on 8 November 2021)) and normalized to the Vinculin loading control.

### 4.12. Luciferase Assay

A MAP2K1 3′-UTR segment of 1009 bp, a MAPK3 3′-UTR segment of 716 bp, and a CRAF 3′-UTR segment of 1017 bp were amplified by PCR from human genomic DNA and inserted into the pGL3 basic vector (#E1751 Promega, Madison, WI, USA) by using the XbaI site immediately downstream from the stop codon of luciferase and the FseI site 8 bp downstream the XbaI site. The sets of primers used to generate specific fragments are listed in Table S3 and named MAP2K1 3′UTR, MAPK3 3′UTR, and CRAF 3′UTR. We also generated mutants with deletions of the target site using the QuikChange II XL Site-Directed Mutagenesis Kit (#200522-5 Agilent Technologies, Santa Clara, CA, USA) according to the manufacturer’s instructions. For MAP2K1 and MAPK3, we performed a second mutagenesis on the mutated plasmid in order to destroy another miRNA target site inside the 3′-UTR of the gene. The sets of primers used to generate specific fragments are listed in Table S3.

### 4.13. CellTiter-Glo^®^ Luminescent Cell Viability Assay

The CellTiter-Glo^®^ Luminescent Cell Viability Assay (Promega, Madison, WI, USA) is a homogeneous method to determine the number of viable cells in a culture based on quantitation of the ATP present, which signals the presence of metabolically active cells. The homogeneous assay procedure involves adding a single reagent (CellTiter-Glo^®^ Reagent) directly to cells cultured in serum-supplemented medium. The homogeneous “add–mix–measure” format results in cell lysis and the generation of a luminescent signal proportional to the amount of ATP present. The amount of ATP is directly proportional to the number of cells present in the culture. The CellTiter-Glo^®^ Assay generates a “glow-type” luminescent signal, produced by the luciferase reaction, which has a half-life greater than five hours. The assay was performed in accordance with the manufacturer’s protocols.

### 4.14. Annexin-V Assay

Cells were seeded in 25-cm^2^ flasks and allowed to attach for 24 h before transfection. After 4 h post-transfection, cells were treated with erlotinib at a single concentration of 1 μg/mL for 6 h, and then, a washout was performed according to the experimental schedule. Cells were harvested, washed once in PBS, and incubated with 25-μg/mL Annexin V-FITC in a binding buffer (Bender MedSystems, Vienna, Austria) for 15 min at 37 °C in a humidified atmosphere in the dark. Cells were then washed in PBS and resuspended in binding buffer. Immediately before the flow cytometric analysis, propidium iodide was added to a final concentration of 5 mg/mL in order to distinguish between the total apoptotic cells and necrotic cells. Experiments were carried out three times, and for each sample, 10,000 events were recorded.

### 4.15. Bromodeoxyuridine (BrdU) Assay

Cells were seeded in six-well plates and allowed to attach for 24 h before transfection. After 4 h post-transfection, cells were treated with erlotinib at a concentration of 1 μg/mL for 6 h, and then, washout was performed according to the experimental schedule. At each time point, they were incubated with a 60-μM Bromodeoxyuridine (BrdU) solution. Cells were then centrifuged and fixed with cold 70% absolute ethanol. The day after, the cells were washed in PBS 1× and incubated with, first, HCL 2 M and then sodium tetraborate and Tween 20 0.5% + BSA 1%. Finally, the cells were incubated with a 1:50 dilution of anti-BrdU antibody (Thermo Fisher Scientific, Waltham, MA, USA) for 1 h and then with a FITC-conjugated secondary antibody (Dako, Agilent Technologies, Santa Clara, CA, USA) diluted 1:250 for 1 h. After antibody incubation, the cells were washed with PBS 1×, stained with propidium iodide solution, and incubated for 2 h at 4 °C before flow cytometry.

### 4.16. Animal Models

All in vivo experiments were performed according to the protocols approved by the Animal Care and Usage Committee of Children’s Hospital Los Angeles (CHLA IACUC protocol #354-16 approved on 23 November 2016). Utilized were 30 NOD scid gamma (NSG) mice (50% male, 50% female equally distributed among the experimental groups) at an age of five to eight weeks, which were bred in-house, genotyped for colony maintenance, and housed in a pathogen-free environment. The animals were sub lethally irradiated with 200-cGy total body irradiation (TBI), as previously described [50], and injected subcutaneously the following day with 4 × 10^6^ A549-16LV (Group A, *n* = 15) or A549-LVEV (Group B, *n* = 15). Tumors were palpable in all animals on Day 9 post-injection; at this time, the animals were sorted into the following three treatment subgroups (*n* = 5) for Group A and three treatment subgroups (*n* = 5) for Group B: Dimethyl sulfoxide (DMSO), erlotinib (50 mg/kg), or selumetinib (25 mg/kg in corn oil). Drugs and DMSO were administered at a total volume of 200 μL via oral gavage 5 days per week. Tumor growth was monitored every 3 days by caliper measurements. On Day 41, mice were euthanized; necropsy performed; and the tumors were excised, measured, and photographed. Tumor volumes were determined with the ellipsoid formula: (W2 × L × π)/6, where L is the largest diameter, and W is the perpendicular diameter.

### 4.17. Statistical Analyses

For patient samples, normality distribution of the miR-16 expression value was tested using the Shapiro–Wilk test. The association between the miR-16 expression and type of tissue, normal (N) or tumoral (T), sample was assessed by the Wilcoxon matched-pairs signed-rank test. The same test was applied to the *KRAS* wild-type and *KRAS*-mutated NSCLC subgroups. Among the tumoral samples, the associations between the type of mutation and miR-16 expression values were assessed by the two-sample Mann–Whitney test. Box plots were presented on a log scale. A *p*-value of 0.05 was adopted for all statistical analyses. The statistical analyses were carried out with STATA/SE 14.1 for Windows (Stata Corp LP, College Station, TX, USA).

Data from the qRT-PCR analysis were analyzed with Applied Biosystems 7500 Real-Time PCR System Software, while the relative expression levels were calculated using the comparative C_t_ method (ΔΔC_t_), as described by Livak and Schmittgen [51]. The results obtained from all the experiments were analyzed with GraphPad Prism v.6.01 Software and were expressed as the mean ± standard deviation (SD). Differences between the groups were estimated using Prism GraphPad v.6.01 Software, and a maximum probability level of 0.05 was chosen for statistical significance.

## 5. Conclusions

Our work identified miR-16 as a key modulator of the primary resistance to TKIs in *KRAS*-mutated NSCLC and suggests that the restoration of miR-16 in combination with erlotinib or selumetinib may be a promising approach in the treatment of *KRAS*-mutated NSCLC.

## Data Availability

The data presented in this study are contained within the article or supplementary material.

## Supplementary Materials

The following are available online at www.mdpi.com/article/10.3390/ijms222413357/s1.

## Abbreviations

AKT	AKT serine/threonine kinase
CMS	Medical scientific committee
CRAF	Raf-1 proto-oncogene, serine/threonine kinase
DMSO	Dimethyl sulfoxide
EGFR	Epidermal growth factor receptor
ERL	Erlotinib
FBS	Fetal bovine serum
KRAS	KRAS proto-oncogene, GTPase
LV16	LV16
LVEV	Lentiviral empty vector
MAP2K1	Mitogen-activated protein kinase kinase 1
MAPK	Mitogen-activated protein kinases
MAPK3	Mitogen-activated protein kinase 3
MEK	Mitogen-activated protein kinase kinase
NSCLC	Non-small cell lung cancer
PI3K	Phosphoinositide 3-chinasi
PMSF	Phenylmethylsulphonyl fluoride
Pre-miRNA	miRNA precursor
SCR	Scrambled negative control
SEL	Selumetinib
TKIs	Tyrosine kinase inhibitors
