# Supplementary material for: MicroRNA-16 Restores Sensitivity to Tyrosine Kinase Inhibitors and Outperforms MEK Inhibitors in KRAS-Mutated Non-Small Cell Lung Cancer
Source: Int J Mol Sci. 2021 Dec 12;22(24):13357. doi: 10.3390/ijms222413357 (PMC8705178; doi:10.3390/ijms222413357)
Supplement: Supplementary file 1 [file ijms-22-13357-s001.zip › ijms-1477852-supplementary.pdf]

SUPPLEMENTARY TABLES

Table S1 KRAS mutation status in the lung adenocarcinoma cell lines used in this study.

Cell line	Mutation type	Nucleotide substitution	Amino acid substitution
A549	Transition	GGT > AGT; G34A	G12S
NCI-H2009	Transversion	GGT > GCT; G35C	G12A

Table S2 Histological and *KRAS* status of NSCLC patients used in this study.

	SAMPLE	HISTOLOGY	KRAS STATUS	KRAS MUTATION
1	L4T	Carcinoid	wt	
2	L5T	Squamous	wt	
3	L9T	Squamous	wt	
4	L10T	Squamous	wt	
5	L11T	Squamous	wt	
6	L12T	Bronchoalveolar Adenocarcinomarcinoma	wt	
7	L13T	Bronchoalveolar Adenocarcinomarcinoma	wt	
8	L18T	Carcinoid	wt	
9	*L19T*	*Adenocarcinomarcinoma*	*mut*	*G34T [G12C]*
10	L20T	Squamous	wt	
11	L22T	Squamous	wt	
12	L29T	Squamous	wt	
13	*L30T*	*Adenocarcinomarcinoma*	*mut*	*G35A [G12D]*
14	L32T	Squamous	wt	
15	L35T	Squamous	wt	
16	L40T	Adenocarcinomarcinoma	wt	
17	L42T	Squamous	wt	
18	L45T	Adenosquamous	wt	
19	L46T	Squamous	wt	
20	L47T	Bronchoalveolar Adenocarcinomarcinoma	wt	
21	L48T	Bronchoalveolar Adenocarcinomarcinoma	wt	
22	L50T	Adenocarcinomarcinoma	wt	
23	L53T	Squamous	wt	
24	L54T	Squamous	wt	
25	*L57T*	*Squamous*	*mut*	*G35A [G12D]*
26	L60T	Squamous	wt	
27	L61T	Adenocarcinomarcinoma	*mut*	*G37T [G13C]*
28	*L62T*	*Adenocarcinomarcinoma*	*mut*	*G35T [G12V]*
29	L63T	Squamous	wt	
30	*L64T*	*Adenocarcinomarcinoma*	*mut*	*G35T [G12V]*
31	L65T	Adenocarcinomarcinoma	wt	
32	*L66T*	*Adenocarcinomarcinoma*	*mut*	*G34T [G12C]*
33	L67T	Squamous	wt	
34	*L70T*	*Adenocarcinomarcinoma*	*mut*	*G35A [G12D]*
35	L73T	Adenocarcinomarcinoma	wt	
36	L74T	Adenocarcinomarcinoma	wt	
37	*L76T*	*Adenocarcinomarcinoma*	*mut*	*G34T [G12C]*
38	L77T	Squamous	wt	
39	*L78T*	*Adenocarcinomarcinoma*	*mut*	*G35A [G12D]*
40	L82T	Adenocarcinomarcinoma +	wt	
41	L83T	Adenocarcinomarcinoma not confirmed	wt	
42	*L84T*	*Adenocarcinomarcinoma*	*mut*	*G34T [G12C]*
43	L86T	Squamous	wt	
44	*L87T*	*Adenocarcinomarcinoma*	*mut*	*G34T [G12C]*
45	L89T	Adenocarcinomarcinoma	wt	
46	L90T	Adenocarcinomarcinoma	wt	
47	*L91T*	*Adenocarcinomarcinoma*	*mut*	*G34T [G12C]*
48	L92T	Adenocarcinomarcinoma	wt	
49	L93T	Carcinoid	wt	

50	L94T	Adenocarcinoma	wt	
51	L95T	carcinoide	wt	
52	L96T	Squamous	wt	
53	*L99T*	*Adenocarcinoma*	*mut*	*G34T [G12C]*
54	L100T	Adenocarcinoma	wt	
55	*L101T*	*Bronchoalveolar Carcinoid*	*mut*	*G35T [G12V]*
56	L102T	Carcinoid	wt	
57	L103T	Large cells	wt	
58	L104T	Adenocarcinoma	wt	
59	L105T	Adenocarcinoma	wt	
60	*L106T*	*Adenocarcinoma*	*mut*	*G34A [G12S]*
61	*L107T*	*Adenocarcinoma*	*mut*	*G35T [G12V]*
62	L109T	Adenocarcinoma	wt	
63	L110T	Adenocarcinoma	wt	
64	L111T	Adenocarcinoma	wt	
65	L112T	Squamous	wt	
66	L113T	Adenocarcinoma	wt	
67	L114T	Adenocarcinoma	wt	
68	L117T	Adenocarcinoma	wt	
69	*L118T*	*Large cells*	*mut*	*G34T [G12C]*
70	L120T	Squamous	wt	
71	L121T	Suqmaous	wt	
72	L122T	Adenocarcinoma	wt	
73	L123T	Squamous	wt	
74	L124T	Squamous	wt	
75	L125T	Squamous	wt	
76	L127T	Adenocarcinoma	wt	
77	L129T	Adenocarcinoma	wt	
78	L130T	Neuroendocrine Tumor	wt	
79	L131T	Adenocarcinoma	wt	
80	L133T	Adenocarcinoma	wt	
81	L134T	Adenocarcinoma	wt	
82	L136T	Adenocarcinoma	wt	
83	L137T	Squamous	wt	
84	L138T	Squamous	wt	
85	L139T	Adenocarcinoma	wt	
86	L140T	Adenocarcinoma	wt	
87	L141T	Epidermoid	wt	
88	*L142T*	*Adenocarcinoma*	*mut*	*G38A [G13D]*
89	L143T	Squamous	wt	
90	L144T	NA	wt	
91	L145T	Adenocarcinoma	wt	
92	L146T	Adenocarcinoma	wt	

Table S3 List of primers used in this study. List of the primers used for PCR, sequencing, plasmid cloning, and plasmid mutagenesis. Underlined sequences indicate the endonuclease restriction site.

Procedure	Primer Name	Froward Primer	Reverse Primer
PCR and Sequencing	k-ras exon 2	GGTGAGTTTGTATTAAAAGGTACTGG	GGTCCTGCACCAGTAATATGC
	189bp 12_13 KRAS	TCATTATTTTTATTATAAGGCCTGCTGAA	CAAAGACTGGTCCTGCACCAGTA
	92bp 12_13 KRAS	TTATAAGGCCTGCTGAAAATGACTGAA	TGAATTAGCTGTATCGTCAAGGCACT
	SEQ MAP2K1 15a_16	GGTGAATGTGGGTAGTCATTC	GTATCCCACACAGTATACTG
	SEQ MAP2K1 15a_16 (Primer3)	GTGGGTAGTCATTCTTACAA	
	pGL3 universal		CACTGCATTTAGTTGTGG
	SEQ MAP2K1 16 (II)	AGAACACAGCATGTGCCAAG	TCTCACAAGGCTCCCTCCTA
	SEQ MAPK3 15a_16	GTTAGAAAATGGACACTGTGC	GGATGCTAGCCCTTGAGACT
	SEQ MAPK3 15a_16 (II)	GCTACACGCAGTTGCAGTACATCG	GATGATACAATTCAGGTCCTCCTG
	SEQ c-RAF 15a_16	AGTTGACTTTGCACCTGTCT	GCCTTGAGCATGGGGAATGT
Cloning	MAP2K1 3’UTR	GCTCTAGAGCTAGCCTTAACCAGCCCAG	GCGGCCGGCCCTCGAGTGAAAAGCTTACCA
	MAPK3 3’UTR	GCTCTAGACCTCTTCTGCCTGCTTCTCT	GCGGCCGGCCCTCGAGGATATCCCTAGGAA
	CRAF 3’UTR	GCTCTAGAGCTAGCCCCACACTGAGGAT	GCGGCCGGCCCTCGAGTTAAAAGGAGGTAA
Mutagenesis	MAP2K1 15a_16	GGCAGTGCATGTGAAGCATGCTTTGAAAATGAGC	GCTCATTTTCAAAGCATGCTTCACATGCACTGCC
	MAP2K1 16 (II)	GGATTGGCTTTGTGCTTGGGGCGATGATCAAAACCTGTGCC	GGCACAGGTTTTGATCATCGCCCCAAGCACAAAGCCAATCC
	MAPK3 15a_16	CCAGTTCAATCTCCCGCGCGCCCTTACCTTCC	GGAAGGTAAGGGCGCGCGGGAGATTGAACTGG
	MAPK3 15a_16 (II)	CCTCAAGTACATCCACTCCGAGATCTAAAGCCC	GGGCTTTAGATCTCGGAGTGGATGTACTTGAGG
	c-RAF 15a_16	CATGTTTTCAGAGAAGCAGGACCTTCTAGACTGC	GCAGTCTAGAAGGTCCTGCTTCTCTGAAAACATG

SUPPLEMENTARY FIGURES

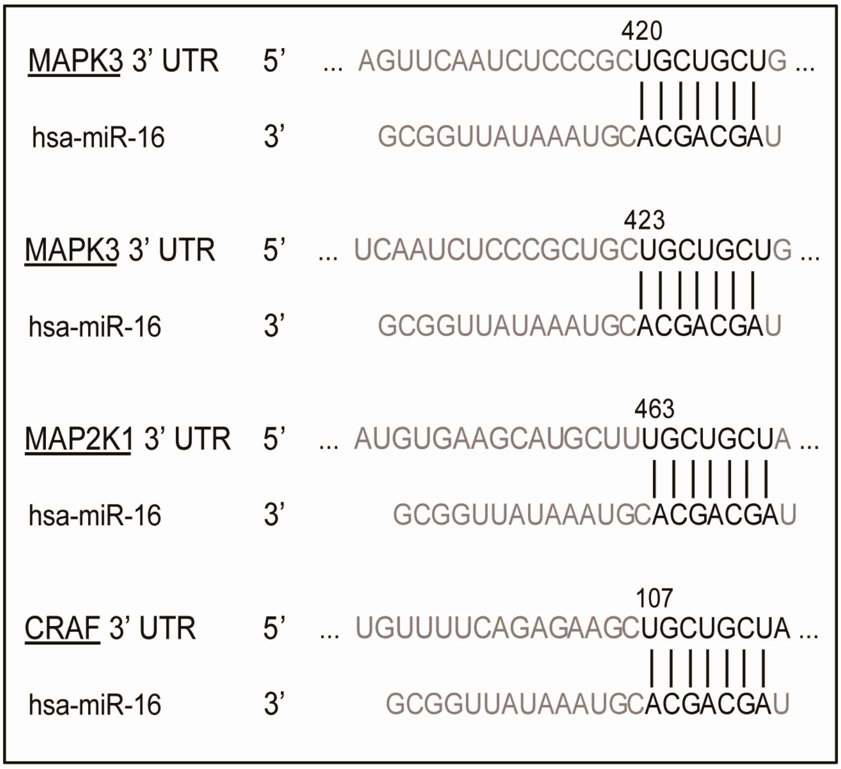

Figure S1 Predicted pairing of *MAPK3*, *MAP2K1*, and *CRAF* 3'UTR region and miR-16. Schematic representation of the predicted miR-16 target sites in the *MAPK3*, *MAP2K1*, and *CRAF* 3'UTR mRNA, according to the Software TargetScanHuman Release v6.2.

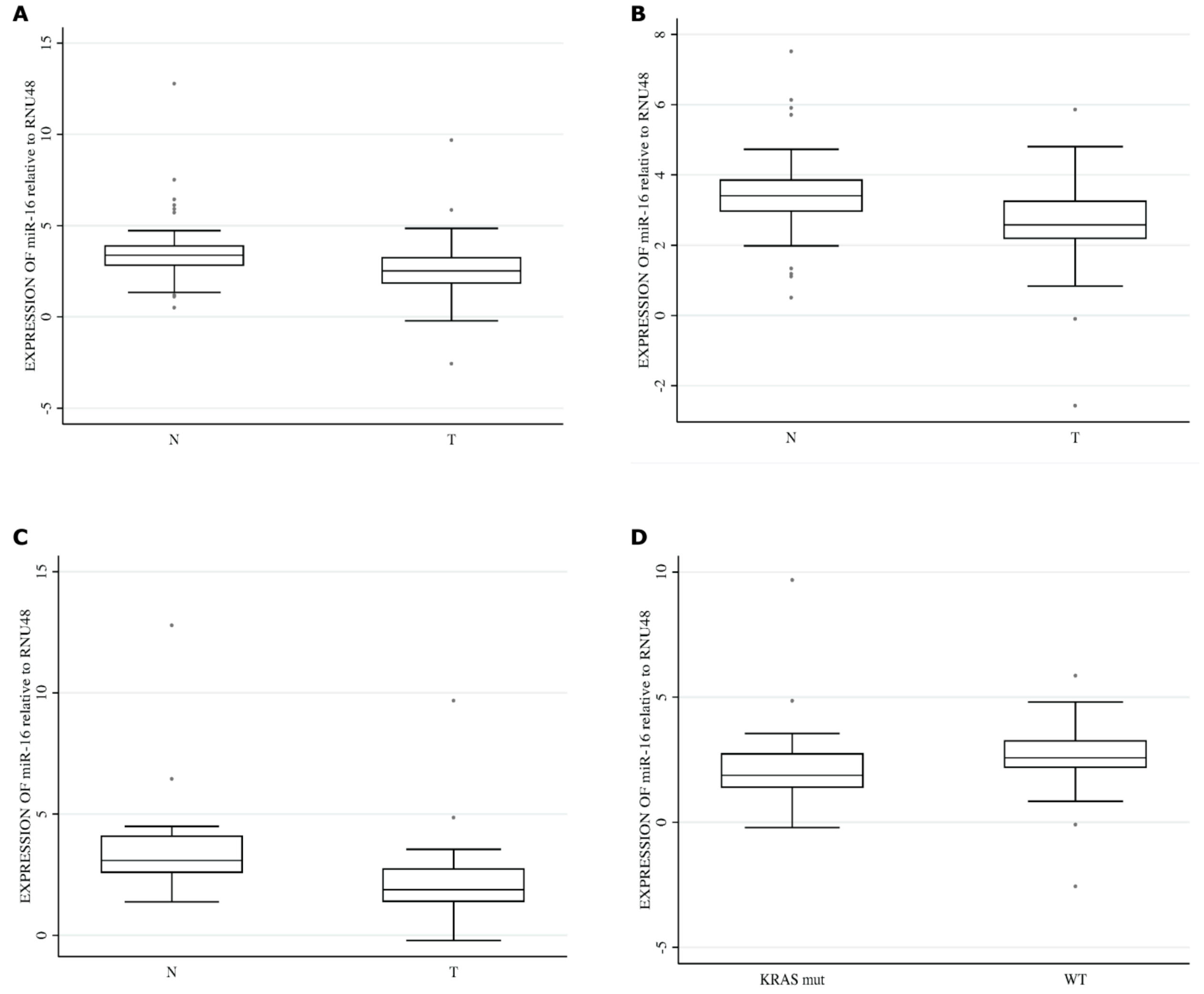

Figure S2 MiR-16 is downregulated in NSCLC primary samples.

(**A** to **D**) Box-plot representation of the miR-16 qRT-PCR expression data in (A) adjacent normal (N) *vs* tumoral (T) samples for all patients examined (*n*=92), (B) in the subset of *KRAS* wild-type samples (*n*=73), (C) in the subset of *KRAS* mutated samples (*n*=19), (D) in the subset of tumoral samples, *KRAS* wild-type (WT) *vs KRAS* mutated (MUT) (*n*=19 and *n*=73, respectively). Wilcoxon matched-pairs signed-rank test in (A) to (D). (A) $P < 0.0001$, (B) $P < 0.0001$, (C) $P = 0.0025$, (D) $P = 0.0107$. Box-plots have been presented on a log-scale.

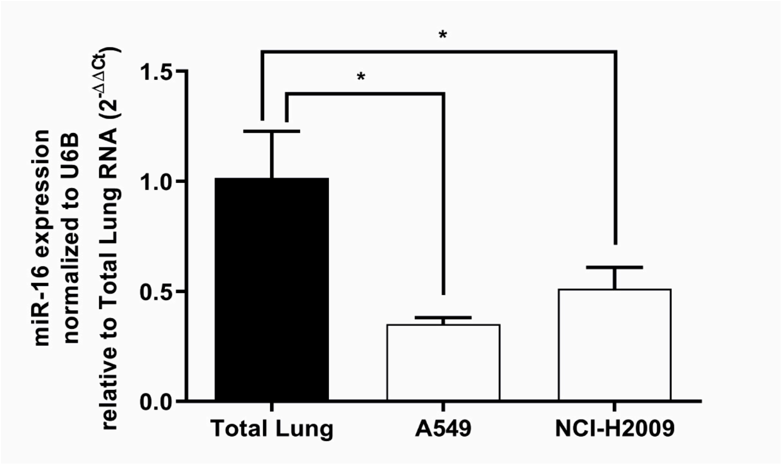

Figure S3 MiR-16 endogenous level in *KRAS* mutated cell lines. Quantitative Real-Time PCR expression analysis of miR-16 endogenous level in *KRAS* mutated cell lines A549 and NCI-H2009, compared to Total Lung RNA (#540019, Agilent Technologies). All data represent means ± SD. * $P < 0.05$. One-way ANOVA.

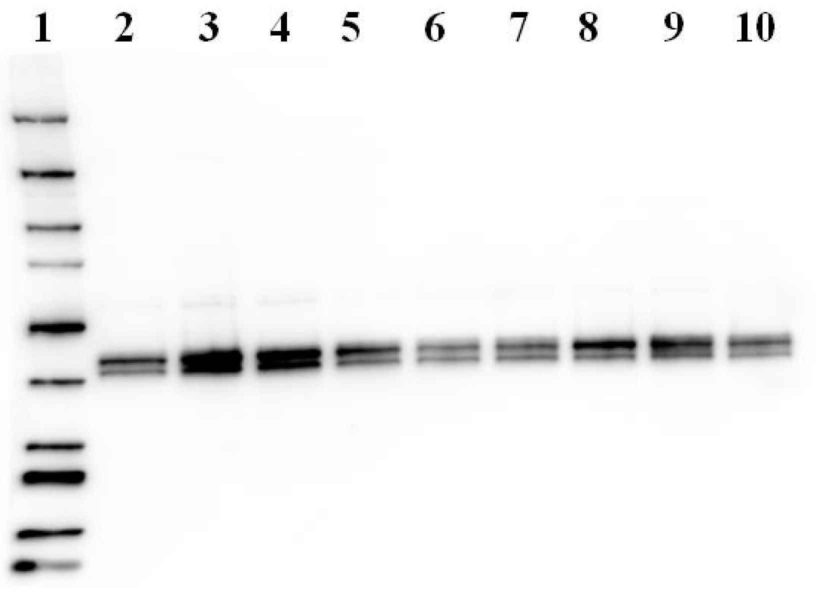

Figure S4 Uncropped western blotting for MAPK3 bands reported in Figure 1B in A549 cells. Lines 5 and 7 refer to A549 cells transfected with scrambled or miR-16 at the conditions reported in Fig. 1B, respectively.

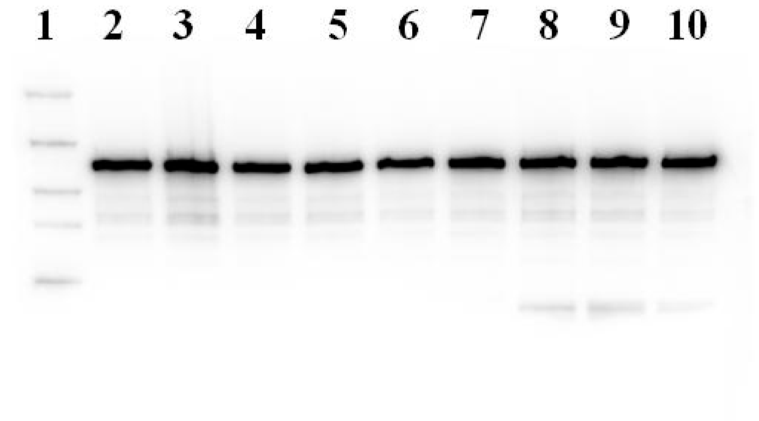

Figure S5 Uncropped western blotting for Vinculin bands reported in Figure 1B in A549 cells. Lines 5 and 7 refer to A549 cells transfected with scrambled or miR-16 at the conditions reported in Fig. 1B, respectively.

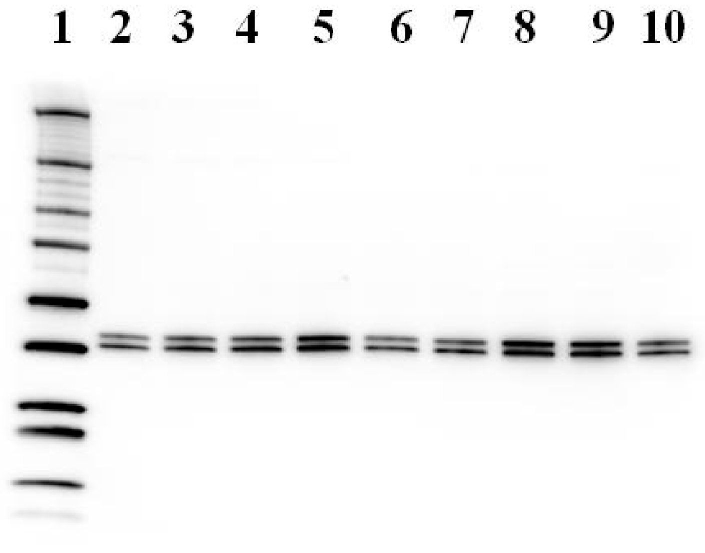

Figure S6 Uncropped western blotting for MAPK3 bands reported in Figure 1B in NCI-H2009 cells. Lines 5 and 7 refer to NCI-H2009 cells transfected with scrambled or miR-16 at the conditions reported in Fig. 1B, respectively.

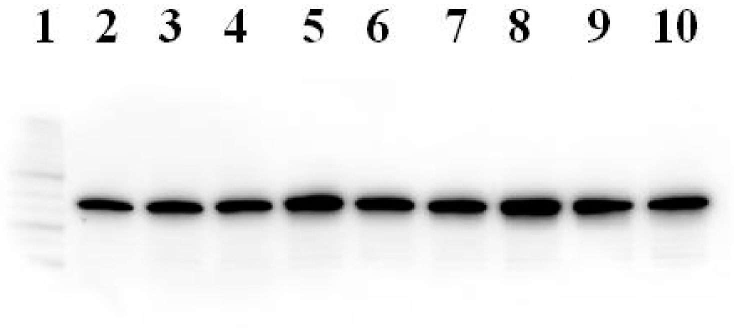

Figure S7 Uncropped western blotting for Vinculin bands reported in Figure 1B in NCI-H2009 cells. Lines 5 and 7 refer to NCI-H2009 cells transfected with scrambled or miR-16 at the conditions reported in Fig. 1B, respectively.

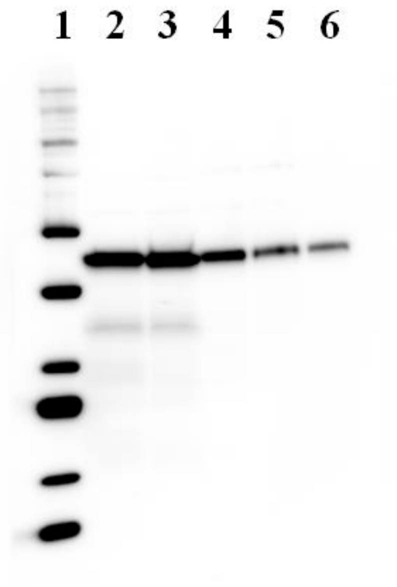

Figure S8 Uncropped western blotting for MAP2K1 bands reported in Figure 1D in A549 cells. Lines 4 and 6 refer to A549 cells transfected with scrambled or miR-16 at the conditions reported in Fig. 1D, respectively.

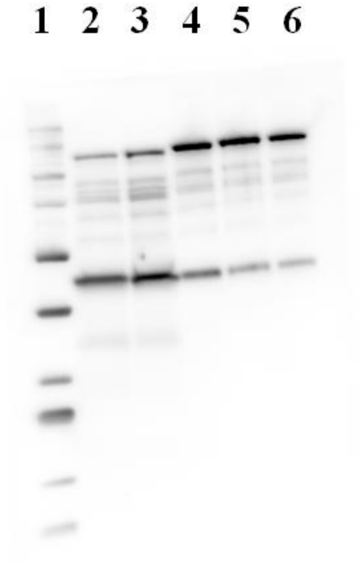

Figure S9 Uncropped western blotting for Vinculin bands reported in Figure 1D in A549 cells. Lines 4 and 6 refer to A549 cells transfected with scrambled or miR-16 at the conditions reported in Fig. 1D, respectively.

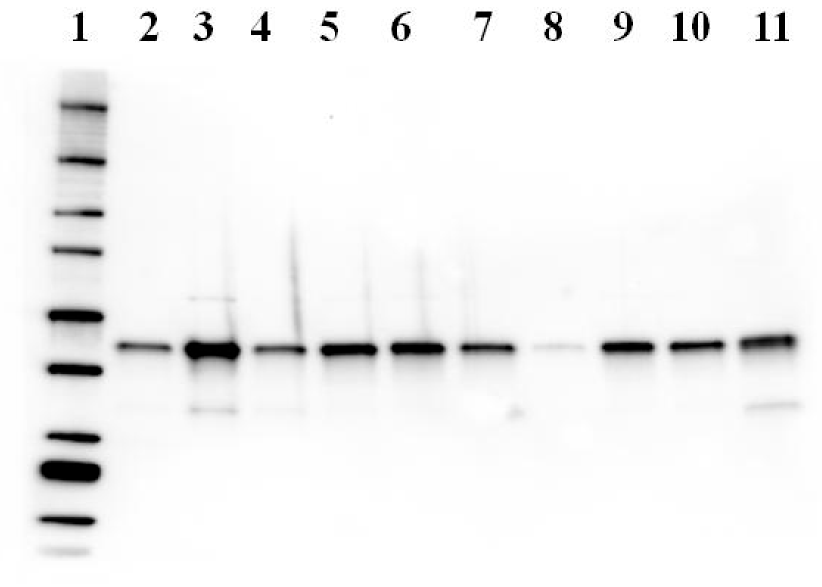

Figure S10 Uncropped western blotting for MAP2K1 bands reported in Figure 1D in NCI-H2009 cells. Lines 6 and 8 refer to NCI-H2009 cells transfected with scrambled or miR-16 at the conditions reported in Fig. 1D, respectively.

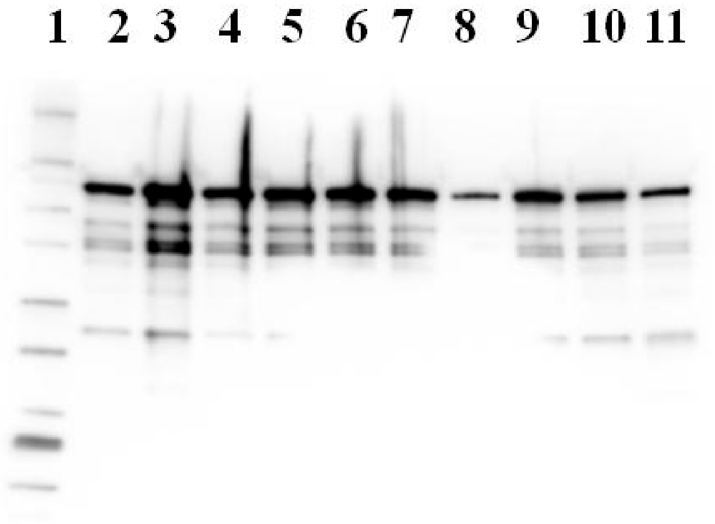

Figure S11 Uncropped western blotting for Vinculin bands reported in Figure 1D in NCI-H2009 cells. Lines 6 and 8 refer to NCI-H2009 cells transfected with scrambled or miR-16 at the conditions reported in Fig. 1D, respectively.

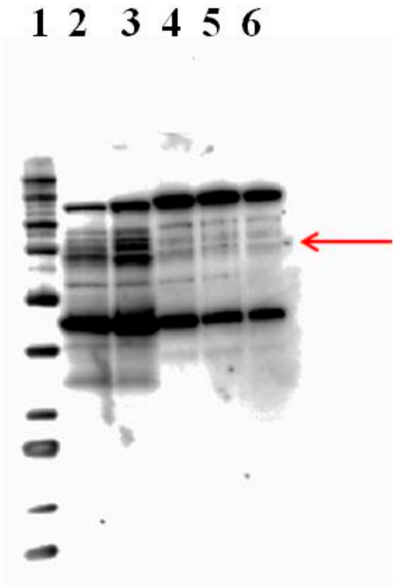

Figure S12 Uncropped western blotting for c-RAF bands reported in Figure 1E in A549 cells. Lines 4 and 6 refer to A549 cells transfected with scrambled or miR-16 at the conditions reported in Fig. 1E, respectively. The red arrow indicates the bands corresponding to c-RAF.

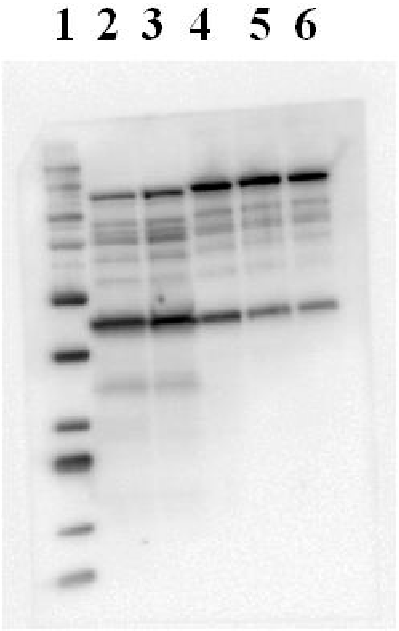

Figure S13 Uncropped western blotting for Vinculin bands reported in Figure 1E in A549 cells. Lines 4 and 6 refer to A549 cells transfected with scrambled or miR-16 at the conditions reported in Fig. 1E, respectively. The red arrow indicates the bands corresponding to Vinculin.

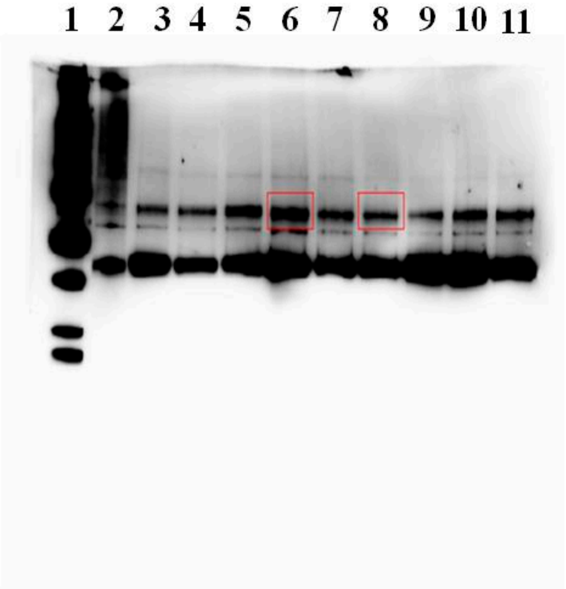

Figure S14 Uncropped western blotting for c-RAF bands reported in Figure 1E in NCI-H2009 cells. Bands within red rectangles refer to NCI-H2009 cells transfected with scrambled (left) or miR-16 (right) at the conditions reported in Fig. 1E, respectively.

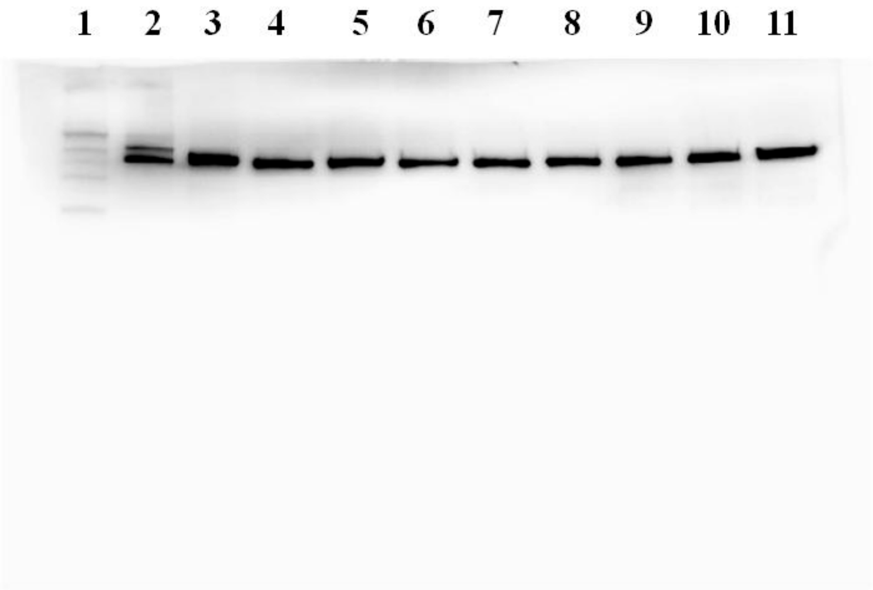

Figure S15 Uncropped western blotting for Vinculin bands reported in Figure 1E in NCI-H2009 cells. Lines 6 and 8 refer to NCI-H2009 cells transfected with scrambled or miR-16 at the conditions reported in Fig. 1E, respectively.

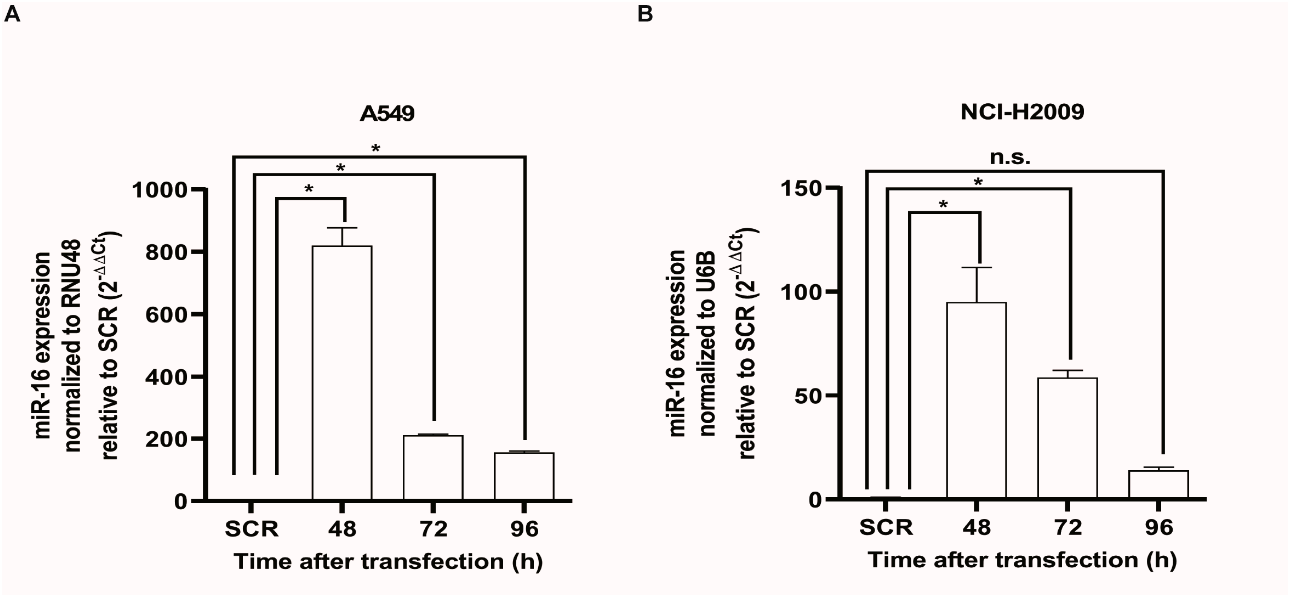

Figure S16 MiR-16 endogenous level in *KRAS* mutated cell lines. (**A** to **B**) Quantitative Real-Time PCR expression analysis of miR-16 levels 48-72-96 h after transfection of A549 (A) and NCI-H2009 (B) cells, compared to scrambled (SCR). All data represent means ± SD. * $P < 0.05$. One-way ANOVA.

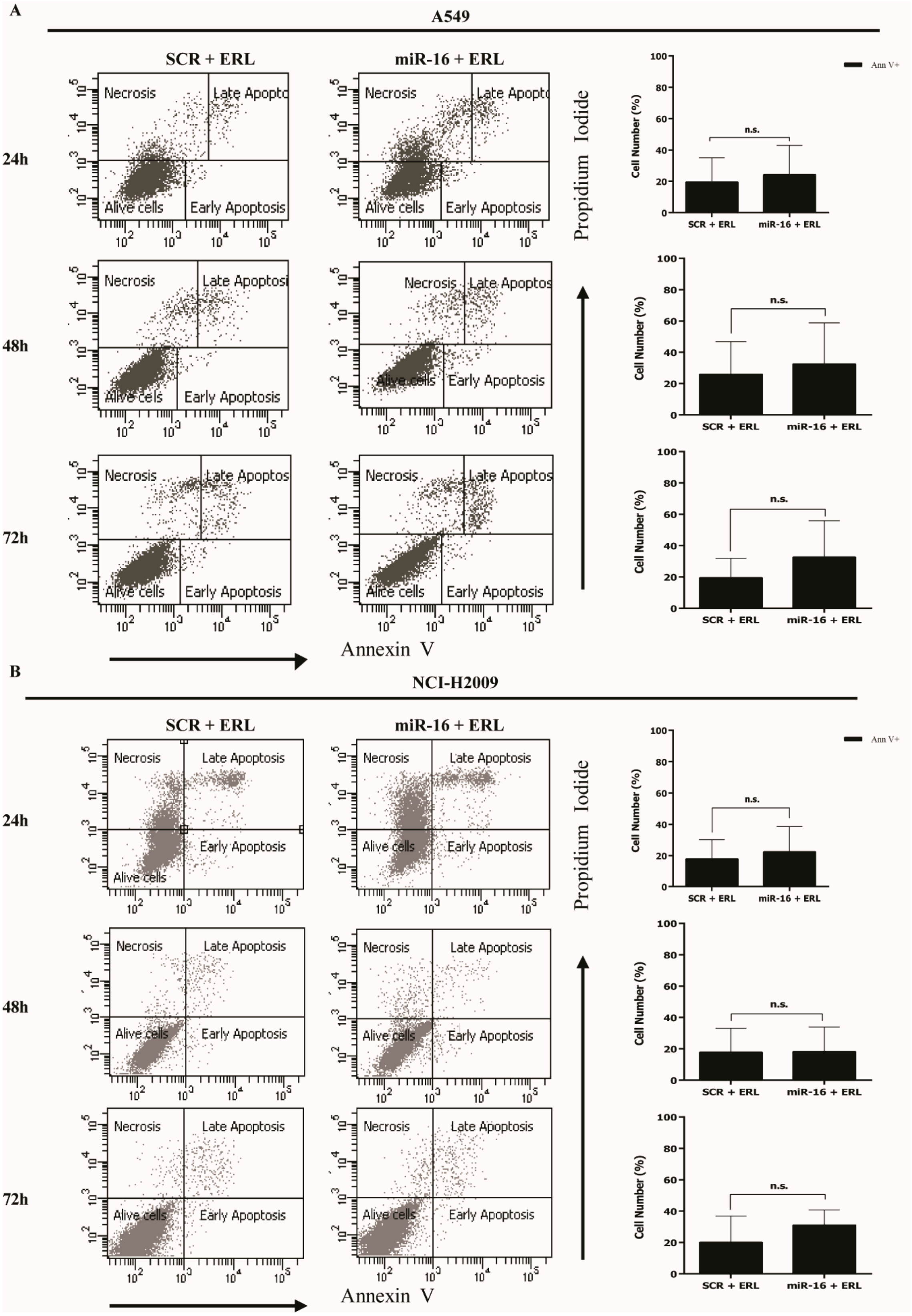

Figure S17 Cytofluorimetric evaluation of erlotinib-induced apoptosis. (**A** and **B**) Representative cytofluorimetric dot plots of apoptotic A549 (A) and NCI-H2009 (B) cells following PI (Propidium Iodide) and Annexin V-FITC co-staining, from one of the three experiments done. The fraction of Annexin V

positive apoptotic cells (Ann V+) was quantified as % of total cells. The average percentage resulting from three different experiments was presented in the histograms on the right, and the Ann V+ fraction was calculated as sum of early and late apoptosis percentages. All data represent means ± SD. n.s.= not significant. Multiple *t* test, corrected for multiple comparison using the Holm-Sidak method was used.

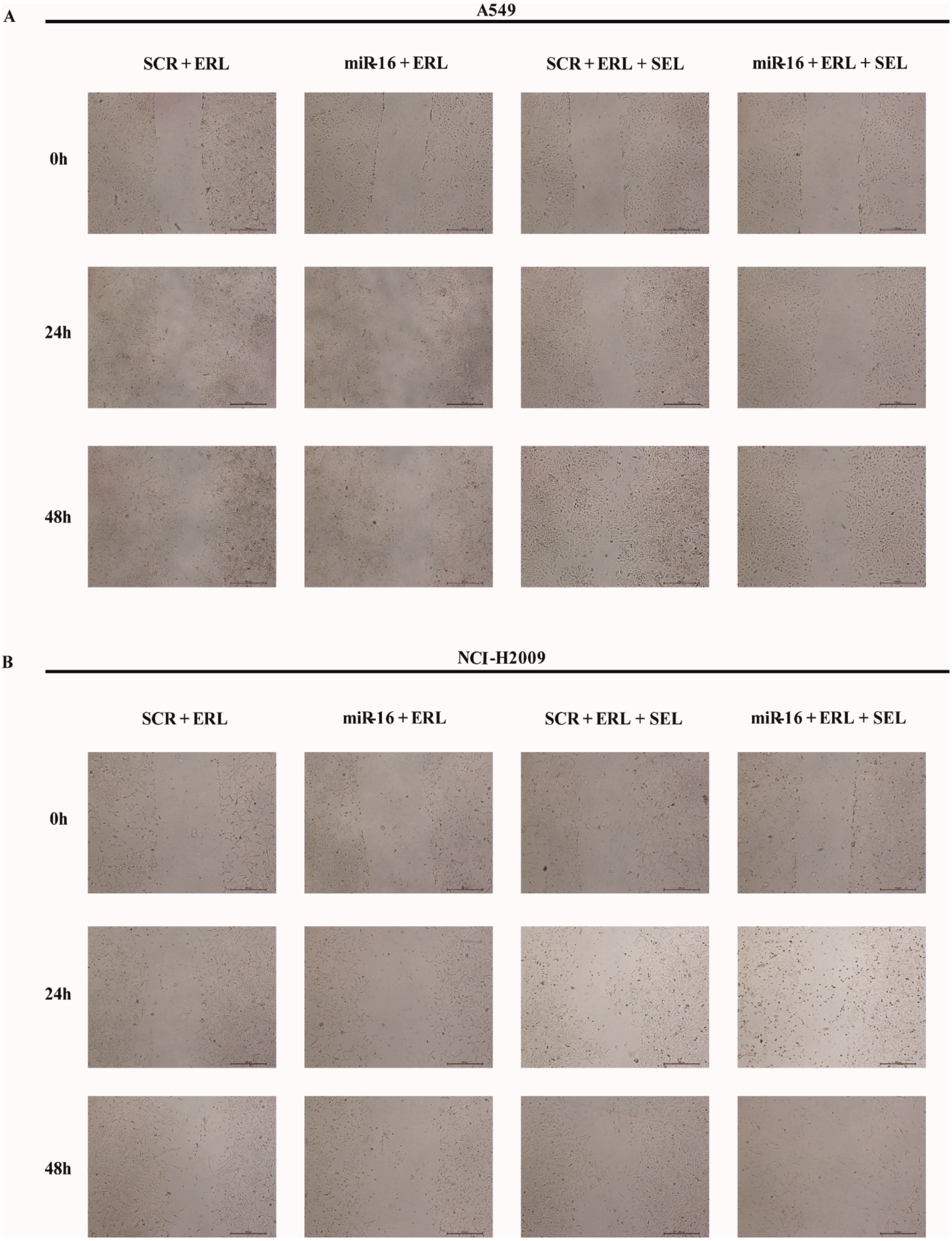

Figure S18 Cell culture wound healing assay. (**A** and **B**) Wounds were generated after cell transfection and drug exposure and cell migration was evaluated during 48h. Representative snapshots at 0, 24 and 48 h are shown and scale bars (500 μm) are added for scratch width measurement.
